# Cross-sectional analysis of CD8 T cell immunity to human herpesvirus 6B

**DOI:** 10.1371/journal.ppat.1006991

**Published:** 2018-04-26

**Authors:** Larissa K. Martin, Alexandra Hollaus, Anna Stahuber, Christoph Hübener, Alessia Fraccaroli, Johanna Tischer, Andrea Schub, Andreas Moosmann

**Affiliations:** 1 DZIF Research Group "Host Control of Viral Latency and Reactivation" (HOCOVLAR), Research Unit Gene Vectors, Helmholtz Zentrum München, Munich, Germany; 2 Department of Obstetrics and Gynecology, University Hospital, LMU Munich, Munich, Germany; 3 Internal Medicine III, Hematopoietic Stem Cell Transplantation, Klinikum der Universität München (LMU), Grosshadern, Munich, Germany; 4 German Center for Infection Research (DZIF–Deutsches Zentrum für Infektionsforschung), Munich, Germany; Blumburg Institute, UNITED STATES

## Abstract

Human herpesvirus 6 (HHV-6) is prevalent in healthy persons, causes disease in immunosuppressed carriers, and may be involved in autoimmune disease. Cytotoxic CD8 T cells are probably important for effective control of infection. However, the HHV-6-specific CD8 T cell repertoire is largely uncharacterized. Therefore, we undertook a virus-wide analysis of CD8 T cell responses to HHV-6. We used a simple anchor motif-based algorithm (SAMBA) to identify 299 epitope candidates potentially presented by the HLA class I molecule B*08:01. Candidates were found in 77 of 98 unique HHV-6B proteins. From peptide-expanded T cell lines, we obtained CD8 T cell clones against 20 candidates. We tested whether T cell clones recognized HHV-6-infected cells. This was the case for 16 epitopes derived from 12 proteins from all phases of the viral replication cycle. Epitopes were enriched in certain amino acids flanking the peptide. Ex vivo analysis of eight healthy donors with HLA-peptide multimers showed that the strongest responses were directed against an epitope from IE-2, with a median frequency of 0.09% of CD8 T cells. Reconstitution of T cells specific for this and other HHV-6 epitopes was also observed after allogeneic hematopoietic stem cell transplantation. We conclude that HHV-6 induces CD8 T cell responses against multiple antigens of diverse functional classes. Most antigens against which CD8 T cells can be raised are presented by infected cells. Ex vivo multimer staining can directly identify HHV-6-specific T cells. These results will advance development of immune monitoring, adoptive T cell therapy, and vaccines.

## Author summary

This paper deals with the immune response to a very common virus, called human herpesvirus 6 (HHV-6). Most people catch HHV-6 in early childhood, which often leads to a disease known as three-day fever. Later in life, the virus stays in the body, and an active immune response is needed to prevent the virus from multiplying and causing damage. It is suspected that HHV-6 contributes to autoimmune diseases and chronic fatigue. Moreover, patients with severely weakened immune responses, for example after some forms of transplantation, clearly have difficulties controlling HHV-6, which puts them at risk of severe disease and shortens their survival. This can potentially be prevented by giving them HHV-6-specific "killer" CD8 T cells, which are cells of the immune system that destroy body cells harboring the virus. However, little is known so far about such T cells. Here, we describe 16 new structures that CD8 T cells can use to recognize and kill HHV-6-infected cells. We show that very different viral proteins can furnish such structures. We also observe that such T cells are regularly present in healthy people and in transplant patients who control the virus. Our results will help develop therapies of disease due to HHV-6.

## Introduction

Human herpesvirus 6 (HHV-6) may be among the most prevalent persistent viruses in the human population. Antibodies to HHV-6 are present in 95–100% of healthy adults [1,2]. Like other herpesviruses, HHV-6 establishes a lifelong infection. HHV-6 is a group of two virus species known as HHV-6A and HHV-6B. Primary infection with HHV-6B, the more widespread species of the two, usually occurs before two years of age, and often causes a common childhood disease known as three-day fever or exanthema subitum [3,4]. The first infection with HHV-6A is thought to occur later and appears mostly asymptomatic [5].

Later in life, HHV-6 may be involved in a variety of diseases. HHV-6A is suspected of contributing to the pathogenesis of thyreoiditis Hashimoto [6] and to neuroinflammatory diseases such as multiple sclerosis [7]. HHV-6B is related to severe complications in immunocompromised patients. After allogeneic hematopoietic stem cell transplantation (allo-HSCT), HHV-6B reactivation is associated with increased all-cause mortality, delayed engraftment, graft-versus-host disease, and damaging infection of the central nervous system [8,9]. Since no HHV-6-specific antiviral agents are available, treatment of infection after allo-HSCT usually involves drugs approved for use against cytomegalovirus (CMV), but these come along with significant side effects such as kidney failure or bone marrow depression [5]. A potentially more efficacious and tolerable form of therapy aims at restoring antiviral T cell immunity, which is defective in patients who reactivate HHV-6 [10]. For other viral infections after allo-HSCT, many clinical investigations have shown that adoptive transfer of donor-derived virus-specific T cells is safe and effective [11]. Most of these studies focused on the herpesviruses CMV and Epstein-Barr virus (EBV), but some have recently included HHV-6-specific T cells [12].

Further development of such immunotherapies and of HHV-6 vaccines will require a detailed understanding of the virus-specific T cell response in health and disease. Information on HHV-6-specific T cell responses is still limited, in particular regarding CD8 T cells [13]. It was shown early that healthy virus carriers have CD4 T cells that respond to HHV-6 lysate or infected cells [14,15]. Target antigens and epitopes of the specific CD4 T cell response were identified first in a study on six selected structural proteins [16], and more recently by a proteomic approach that has identified ten viral antigens targeted by CD4 T cells [17]. Information on the targets of CD8 T cells has remained much more limited. Responses to five HHV-6B proteins have been investigated so far, and a number of epitopes from these proteins that are presented by infected cells were identified [18–21]. These proteins were chosen because of their (mostly distant) homology to CMV proteins that elicit CD8 T cell responses. However, HHV-6B encodes approximately 98 unique proteins [22], and the hypothesis remains unproven that T cell responses to HHV-6 and CMV are similarly structured or directed to corresponding antigens. The biological differences between these viruses are significant despite their evolutionary relationship as β-herpesviruses, and widespread cross-reactivity of T cells to HHV-6 and CMV seems unlikely considering that most of their proteins have quite divergent sequences [21]. Individual HHV-6 epitope-specific CD8 T cell responses were described to be of low frequency in peripheral blood [18–21], and it has remained unknown whether stronger responses exist.

These open questions prompted us to devise a method to analyse the CD8 T cell response to HHV-6 in a more comprehensive, cross-sectional fashion. Screens with libraries of peptides have been particularly efficient in obtaining copious information on the CD8 T cell repertoire against complex viruses [23–25]. However, due to the large number of possible targets, each such study has necessarily neglected some aspects of analysis, either regarding antigen coverage, HLA allotype coverage, precision of epitope identification, or verification of T cell function in the context of infection. Since detection of ex vivo responses to artificial peptides is not sufficient to prove the presence of T cells that recognize functional viral epitopes [26], it is of particular importance to verify recognition of infected cells by individual peptide-specific T cells.

To obtain a cross-sectional overview of the truly functional repertoire of HHV-6B-specific CD8 T cells and their target antigens and epitopes, we chose to base our approach on the entirety of HHV-6B proteins, but to focus on only one HLA class I allotype. We considered HLA-B*08:01 to be particularly suitable for such a study, because of the clarity of its peptide anchor motif [27] and its tendency to present dominant CD8 T cell epitopes in human viral infections [23,28–30]. To verify T cell specificity and function, we established specific T cell clones wherever possible, and used these to verify HLA restriction and recognition of infected cells. Our results show that the HHV-6-specific CD8 T cell repertoire targets multiple epitopes from all phases of the viral life cycle. We identify potent epitopes and track them in patients. We discuss implications for improved immune monitoring, studies of viral pathogenesis, and immunotherapy designs.

## Results

### Cross-sectional screen of HHV-6B for CD8 T cell epitopes

We wished to obtain a cross-sectional overview of HHV-6B antigens targeted by CD8 T cells. The reference sequence for HHV-6B strain Z29 contains 98 unique protein-coding genes or annotated ORFs with a total of 43,836 amino acids. For reasons of feasibility, we decided to screen the viral proteome for specific T cells with only one representative HLA class I restriction. We chose HLA-B*08:01, the second most frequent HLA-B allotype in populations of European origin [31,32]. We had two more reasons for this choice. First, T cell responses to HLA-B*08:01-restricted viral epitopes are often among the strongest that are observed in a particular virus. Examples of such epitopes are shown in Table 1. Second, B*08:01-presented peptides recognized by such T cells mostly conform to a clear-cut consensus motif [27,33,34]. This motif demands basic anchor residues (arginine or lysine) in positions 3 and 5 and an aliphatic residue (leucine, isoleucine, valine, or methionine) in the C-terminal position of an octameric or nonameric peptide. The HHV-6B reference sequence (strain Z29, GenBank NC_000898) contains 146 octameric and 153 nonameric peptides with this B*08:01 epitope motif, and 77 of 98 nonredundant ORFs contained at least one candidate (see Supporting S1 Table for a full list). These peptides were synthesized and used in the following experiments.

**Table 1:** Examples of immunodominant HLA-B*08:01-restricted viral epitopes.

	sequence	viral antigen	reference
8-mer epitopes	RA**K**F**K**QL**L**	EBV BZLF1	[35]
	FL**K**E**K**GG**L**	HIV Nef	[28,36]
	LI**R**L**K**PT**L**	HCV NS3	[37]
9-mer epitopes	FL**R**G**R**AYG**L**	EBV EBNA3A	[38,39]
	QA**K**W**R**LQT**L**	EBV EBNA3A	[39,40]
	EL**R**R**K**MMY**M**	CMV IE-1, variant 1	[23]
	EL**K**R**K**MIY**M**	CMV IE-1, variant 2	[23]
	QI**K**V**R**VDM**V**	CMV IE-1	[23]
	HS**K**K**K**CDE**L**	HCV NS3	[41]
	EL**R**S**R**YWA**I**	Influenza NP	[27,34]
	WL**K**I**K**RDY**L**	Vaccinia A50R	[42]
anchor motif	XX**R**X**R**XX(X)**L** ** K K V** ** I** ** M**		

**Fig 1:**
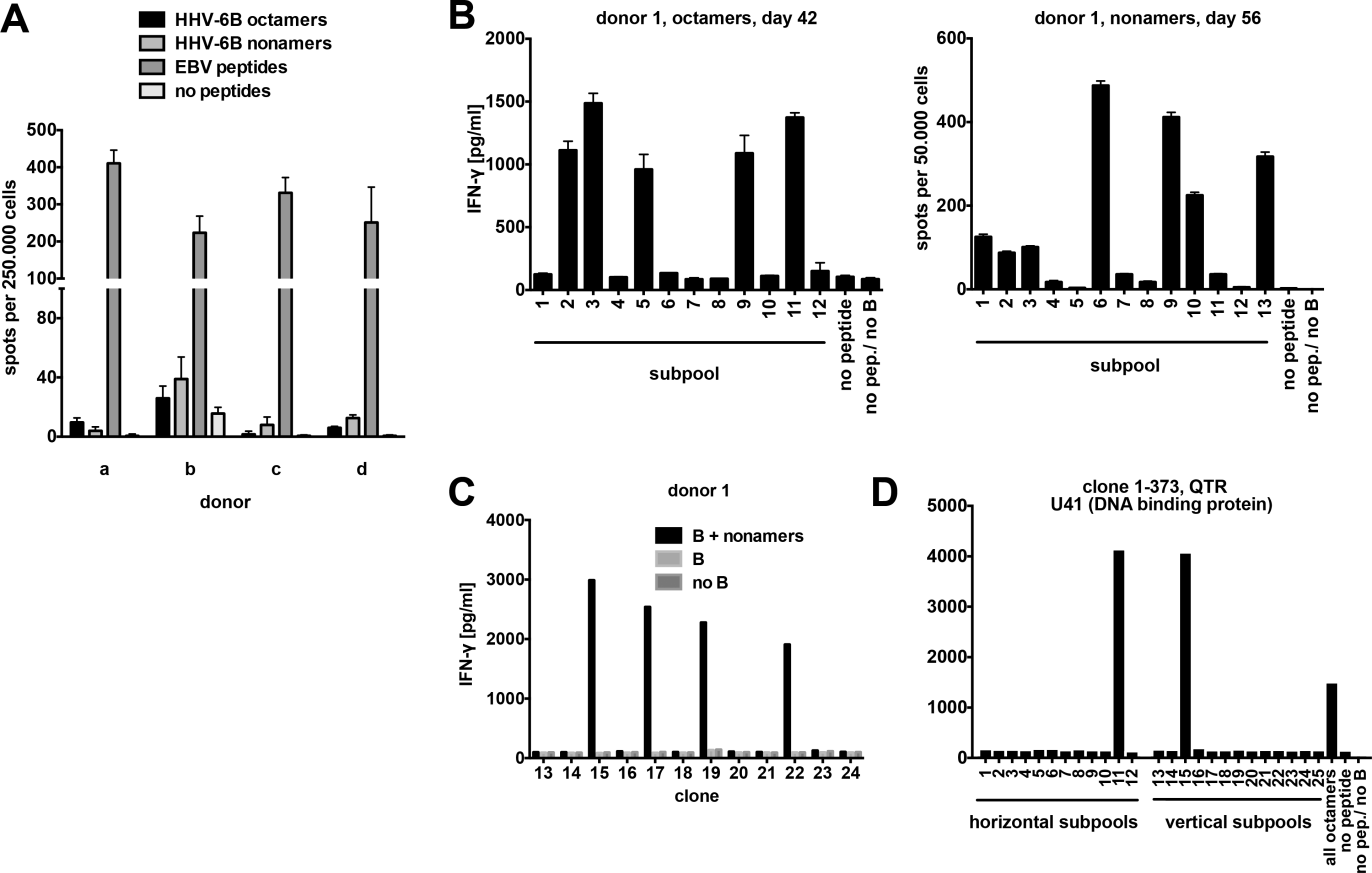
HHV-6B peptide-specific polyclonal T cell lines and CD8 T cell clones. (A) Peptide-specific T cells in PBMCs from four healthy HLA-B*08:01-positive donors (a-d) were detected in an IFN-γ ELISPOT assay. PBMCs were stimulated with pools of 146 octameric or 153 nonameric HHV-6B/HLA-B*08:01 candidate peptides, 29 Epstein-Barr virus peptides (positive control), or no peptides. Mean+SD of 3 replicates is shown. (B) T cell cultures from donor 1 were stimulated with the complete octamer or nonamer peptide pools for 42 or 56 days as indicated, and then tested in IFN-γ ELISA or ELISPOT assays for reactivity to non-overlapping peptide subpools. HLA-B*08:01-positive activated B cells (mini-LCLs) were used to present peptides. Mean+range of 2 replicates is shown. (C) CD8 T cell clones were screened for reactivity to HHV-6B peptide pools in IFN-γ ELISA, as shown in this example for 12 T cell clones. Autologous CD40-activated B cells were used to present peptides. (D) To identify each T cell clone's target within the peptide libraries, T cells were stimulated with "crossed" peptide subpools. One positive signal each for horizontal and vertical subpools identifies the target peptide, as shown here for one T cell clone, which turned out to be specific for the QTR peptide from U41.

First, we attempted to determine the frequency of T cells specific for these HHV-6B-derived peptides in peripheral blood of healthy carriers. We stimulated PBMCs ex vivo with pools of the 146 octameric or the 153 nonameric peptides in an IFN-γ ELISPOT assay. Responses to HHV-6B peptide pools were much weaker than those to an EBV peptide pool, and generally below 1 / 10 000 PBMCs (Fig 1A).

Therefore, we decided to enrich HHV-6B-specific T cells from peripheral blood by peptide stimulation. PBMCs from four B*08:01-positive healthy HHV-6B carriers were initially stimulated with octamer or nonamer peptide mixes, and then restimulated every week with autologous CD40-activated B cells loaded with the same peptide mixes. Fig 1B shows analyses of such cultures from donor 1 after six to eight weeks of cultivation. Specific reactivity was observed against five subpools of the octameric peptides and at least seven subpools of the nonameric peptides, suggesting the presence of T cells specific for at least twelve HHV-6B peptides in this donor.

**Fig 2:**
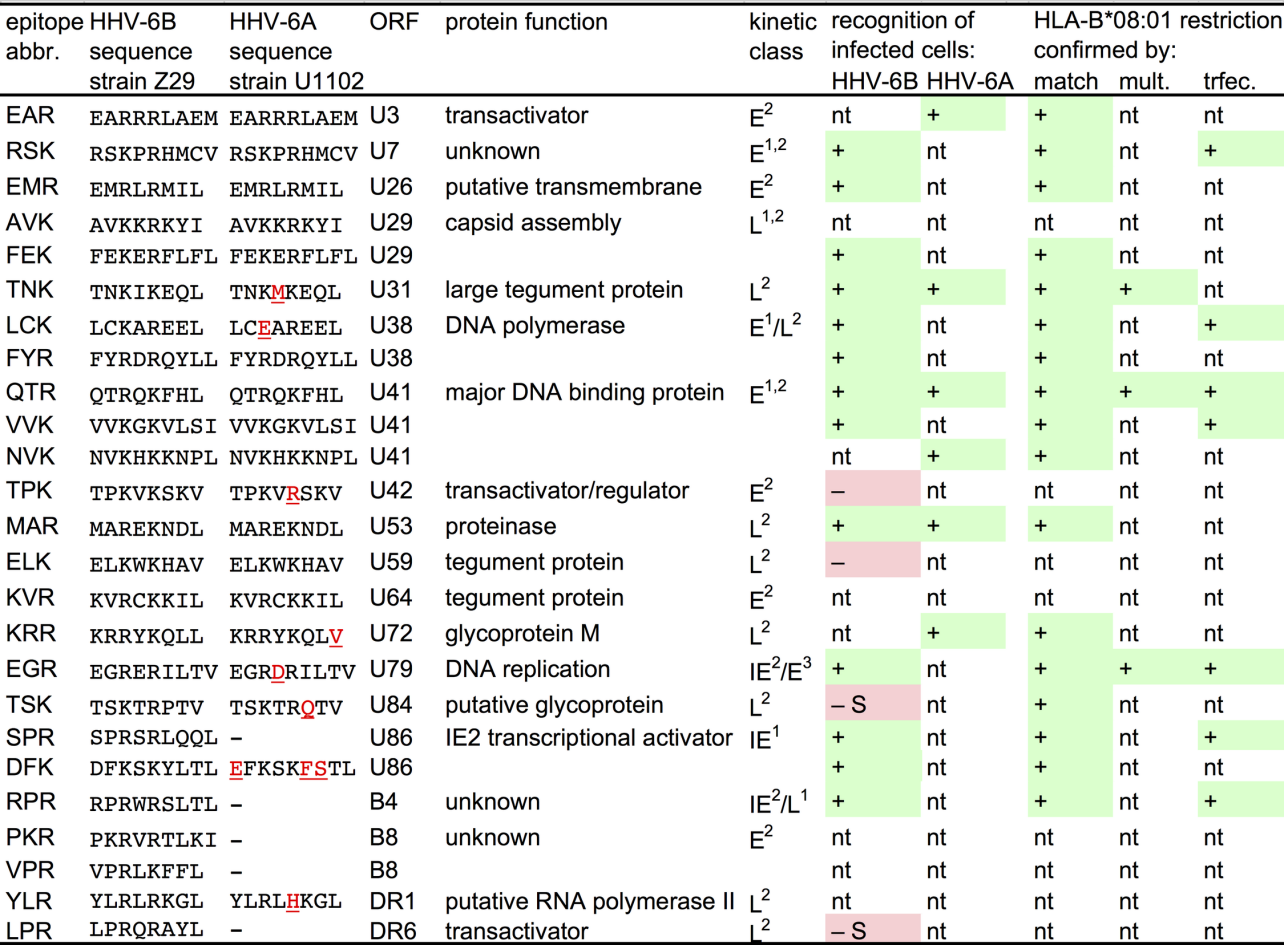
Overview of HLA-B*08:01-restricted peptides and epitopes recognized by CD8 T cells. Each entry corresponds to one or more CD8 T cell clones that recognized the HHV-6B peptide as indicated. Peptides were derived from HHV-6B strain Z29. Amino acid positions that differ between HHV-6B and HHV-6A are underlined. Kinetic classification of antigens follows ^1^Oster et al. [43], ^2^Tsao et al. [44], or ^3^Taniguchi et al. [45]. Recognition of infected cells was analyzed using HHV-6B strain HST and HHV-6A strain U1102. Restriction through HLA-B*08:01 was verified by analyzing reactivity to sets of HLA-matched and mismatched targets ("match"), by HLA/peptide multimer staining ("mult."), or by analyzing recognition of HLA-transfected targets ("trfec."); "nt" means not tested. "–S" indicates that lack of recognition of infected cells may be explained by differences between HHV-6B strains Z29 and HST. In strain HST, the TSK peptide has the sequence TSKTRQTV (the same as in U1102), and the LPR peptide is probably not expressed due to an upstream frameshift mutation [46].

After six to eight weeks of cultivation, limiting dilution of the peptide-stimulated cultures was performed to generate T cell clones. Between 6% and 23% of T cell clones were specific for the HHV-6B peptide pool that was used for expansion (Table 2), as demonstrated by their specific IFN-γ secretion in response to peptide-loaded B cells (Fig 1C). Most of these clones could be sufficiently expanded to determine their precise peptide specificity by testing with peptide subpools (Fig 1D) and individual peptides in IFN-γ ELISA assays. Collectively, T cell clones recognized 25 HHV-6B peptides from 19 proteins or open reading frames (Fig 2).

**Table 2:** Yield of T cell clones specific for HHV-6B peptides.

	total clones tested	HHV-6B peptide-specific clones	minimal no. of distinct specificities
donor 1, octamers	250	37 (15%)	6
donor 2, octamers	300	45 (15%)	10
donor 11, octamers	190	26 (14%)	n.d.
donor 12, octamers	44	10 (23%)	n.d.
donor 1, nonamers	253	47 (19%)	11
donor 2, nonamers	144	8 (6%)	2
donor 11, nonamers	189	24 (13%)	n.d.

**Fig 3:**
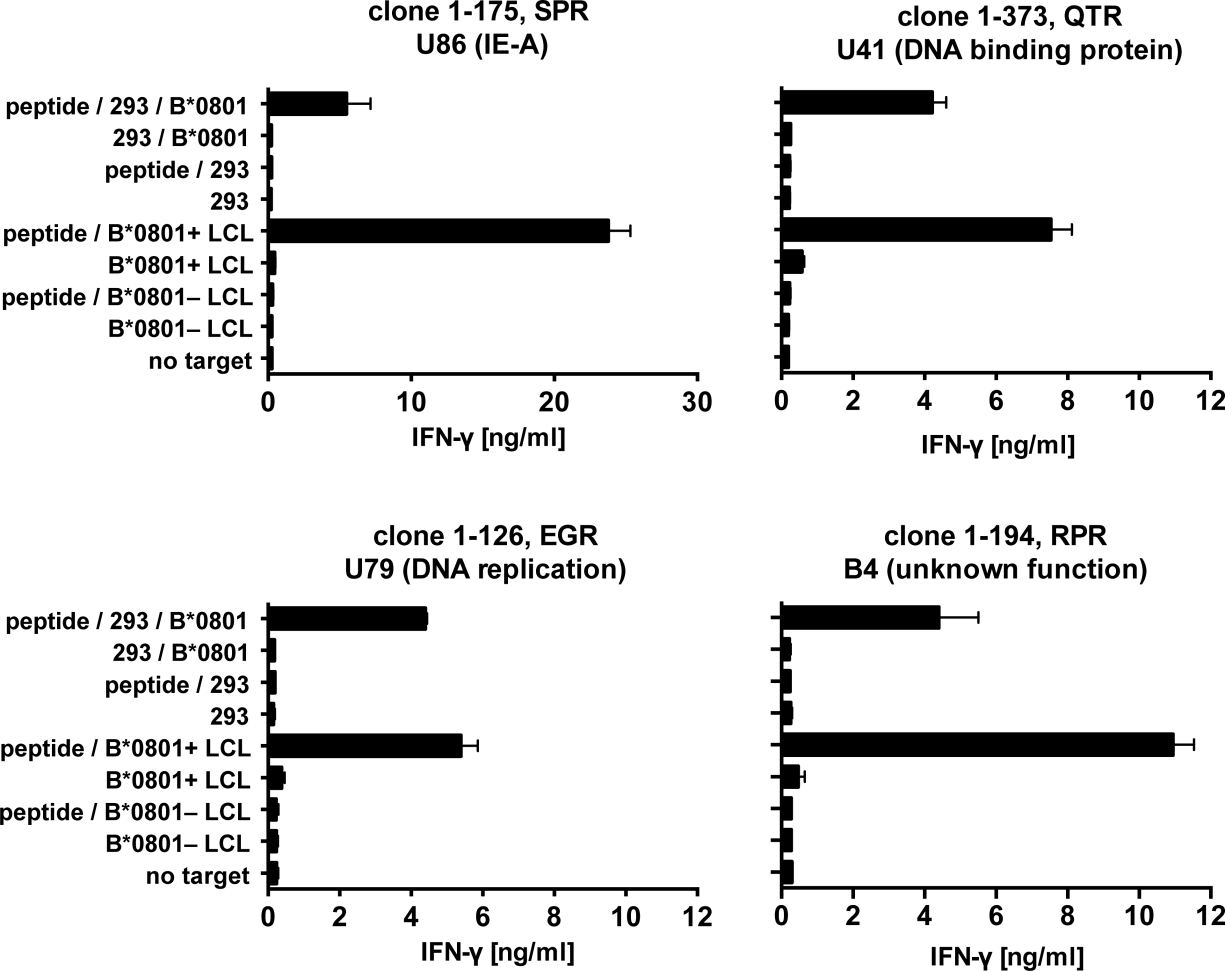
Verification of HLA-B*08:01 restriction of HHV-6B peptides. CD8 T cell clones were tested for specific recognition of 293T kidney cells transiently transfected with HLA-B*08:01 and loaded with the specific cognate peptide. HLA-B*08:01-matched and mismatched LCLs were also tested. IFN-γ was quantified by ELISA. Mean and range of two replicates from one of two experiments is shown. Peptide specificities are indicated by the first three letters of the amino acid sequence (see Fig 2), the gene name, and in parentheses the protein name or function.

For seven specificities, we verified restriction through HLA-B*08:01 by tests with B*08:01-transfected, peptide-loaded 293T cells and, for comparison, with peptide-loaded B*08:01-matched B cells. The very clear patterns of IFN-γ secretion indicated that all T cell clones tested were restricted through HLA-B*08:01, as shown for four clones in Fig 3.

### Infected cells present HLA-B*08:01-restricted epitopes to CD8 T cells

**Fig 4:**
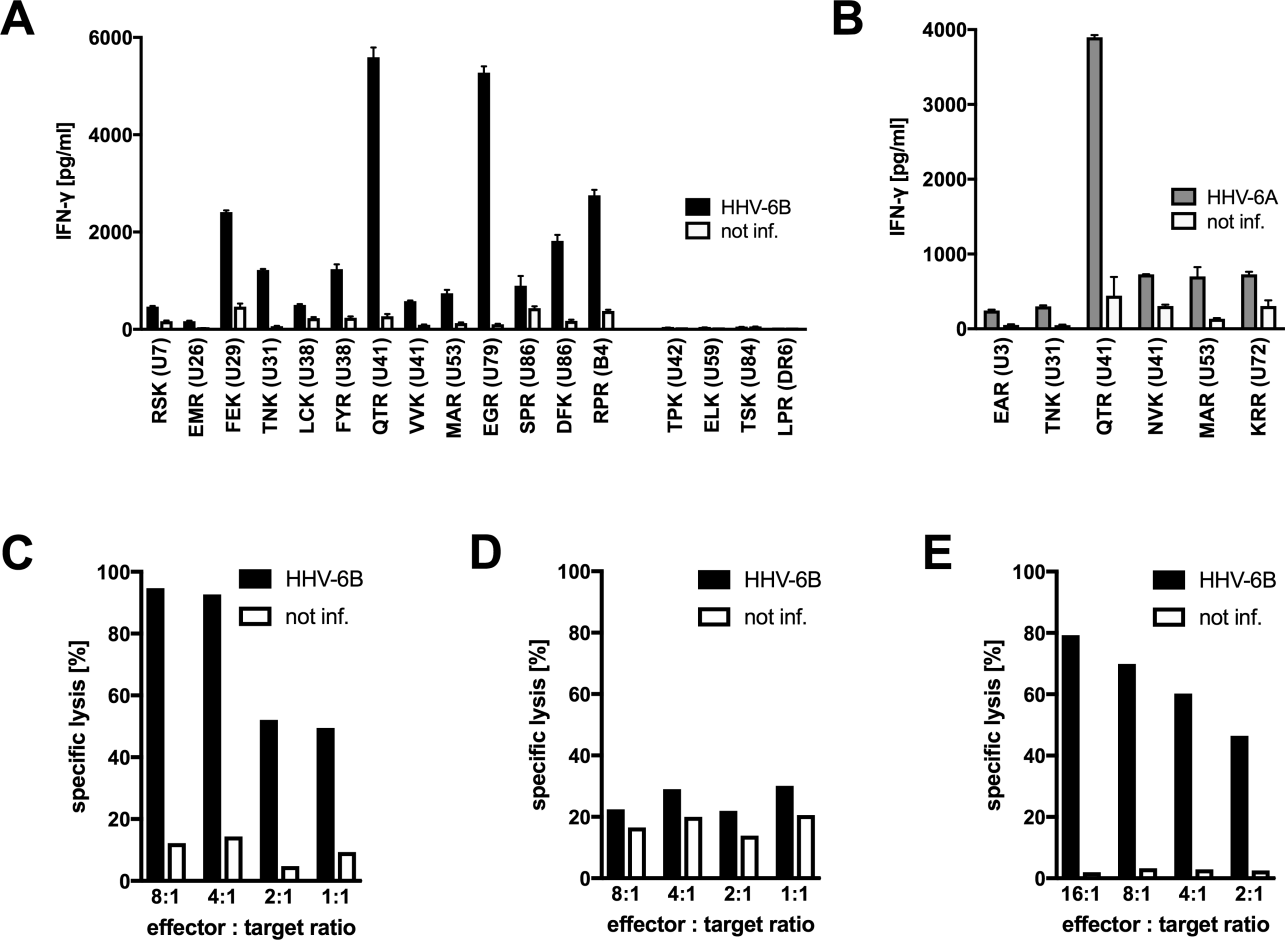
HHV-6-infected cells present HLA-B*08:01-restricted epitopes to CD8 T cells. (A, B) Analysis of IFN-γ secretion in response to infected cells. PHA-activated primary HLA-B*08:01-positive CD4 T cells were infected with HHV-6B strain HST (panel A) or HHV-6A strain U1102 (panel B), or remained uninfected. After six days, infected cells or controls were cocultivated with CD8 T cell clones of diverse specificities. After overnight coincubation, IFN-γ secretion was measured by ELISA. Mean and range of two replicates is shown. (C–E) Cytotoxic activity in response to infected cells. A T cell clone specific for the DFK epitope from U86 (C,D) or a polyclonal DFK-specific T cell line (E), both from donor 5, were tested against primary CD4 T cells infected with HHV-6B strain HST for six days or parallel controls in a calcein release assay. (C, E) HLA-B8-positive targets. (D) HLA-B8-negative targets.

We analyzed whether specific T cell clones were able to recognize their cognate antigen on HHV-6B-infected cells. Primary CD4 T cells from B*08:01-positive donors were activated with phytohemagglutinin (PHA) and infected with HHV-6B strain HST. Infected cultures were combined with peptide-specific CD8 T cell clones to test for specific IFN-γ secretion. T cell clones with 17 peptide specificities could be tested. For 13 of these, we observed specific recognition of HHV-6B infection at day 6, as shown in Fig 4A and summarized in Fig 2. Since most of the HHV-6B peptides recognized by T cells were fully or closely homologous to corresponding sequences in HHV-6A (Fig 2), we also tested the reactivity of T cell clones with six specificities against HHV-6A-infected cells. All six T cell clones recognized infected cells (Fig 4B). For three epitopes, we could demonstrate presentation by both HHV-6B-infected and HHV-6A-infected cells. Three additional specificities could only be tested against HHV-6A, due to limited cell numbers. Four of the six HHV-6A epitopes were identical to their HHV-6B counterparts, two differed in only one conservatively exchanged amino acid. Overall, these experiments demonstrated that 16 of the 25 candidate peptides against which T cell clones could be established (Fig 2) were bona fide epitopes processed and presented by cells infected with HHV-6B or 6A. Four candidates were not recognized, and five candidates could not be tested because T cell clones did not sufficiently expand and survive. We also tested cytotoxic reactivity against HHV-6B-infected target cells, focusing on CD8 T cells specific for the DFK peptide from U86. Both a DFK-specific CD8 T cell clone and a polyclonal CD8 T cell line, obtained by PBMC stimulation with the peptide DFK, displayed strong cytotoxic activity against HHV-6B-infected cells, but not non-infected cells; HLA-B8 expression of the target cells was required for this activity (Fig 4C–4E).

**Fig 5:**
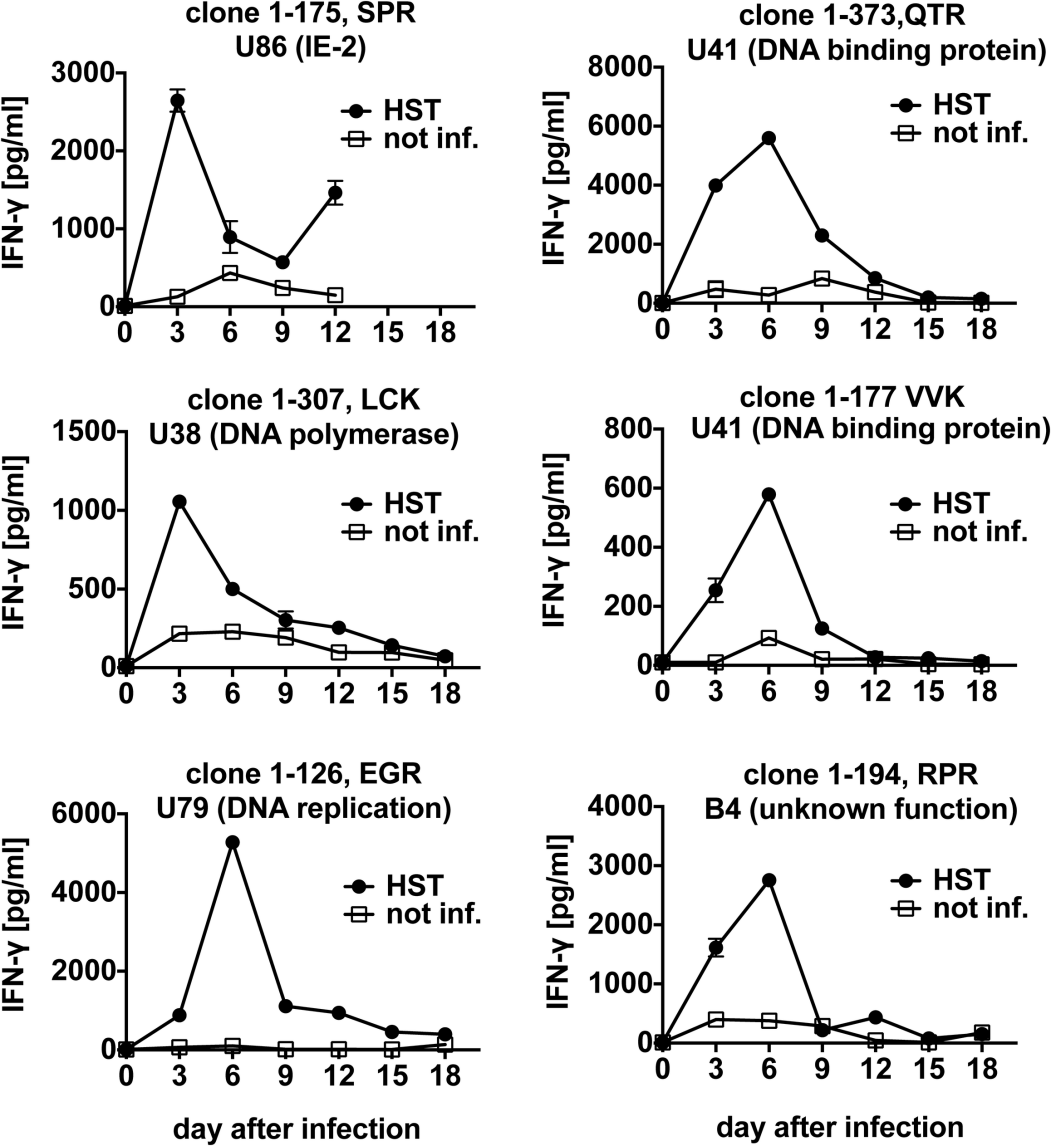
Time course of presentation of HLA-B*08:01-restricted HHV-6B epitopes to CD8 T cells. PHA-activated primary HLA-B*08:01-positive CD4 T cells were infected with HHV-6B strain HST, and cocultivated overnight from the indicated time after infection with CD8 T cell clones of diverse specificities. IFN-γ secretion was measured by ELISA. Mean and range of two replicates from one of three experiments is shown.

We proceeded to analyze recognition of target cells over a period of 12 to 18 days of infection with HHV-6B (Fig 5). Some T cell clones reached a maximum of reactivity at three days of infection, others at six days. Timing of maximal recognition did not appear to correlate with the described expression kinetics of HHV-6B antigens. For example, the SPR epitope from immediate-early (IE) antigen U86 was maximally recognized on day 3, but other IE antigens [44] such as U79 and B4 reached a maximum of recognition on day 6. Presumably, time to completion of antigen processing differed between antigens, or potential secondary cycles of virus production and re-infection within the infected CD4 T cell culture augmented the presentation of some antigens to specific CD8 T cells.

**Fig 6:**
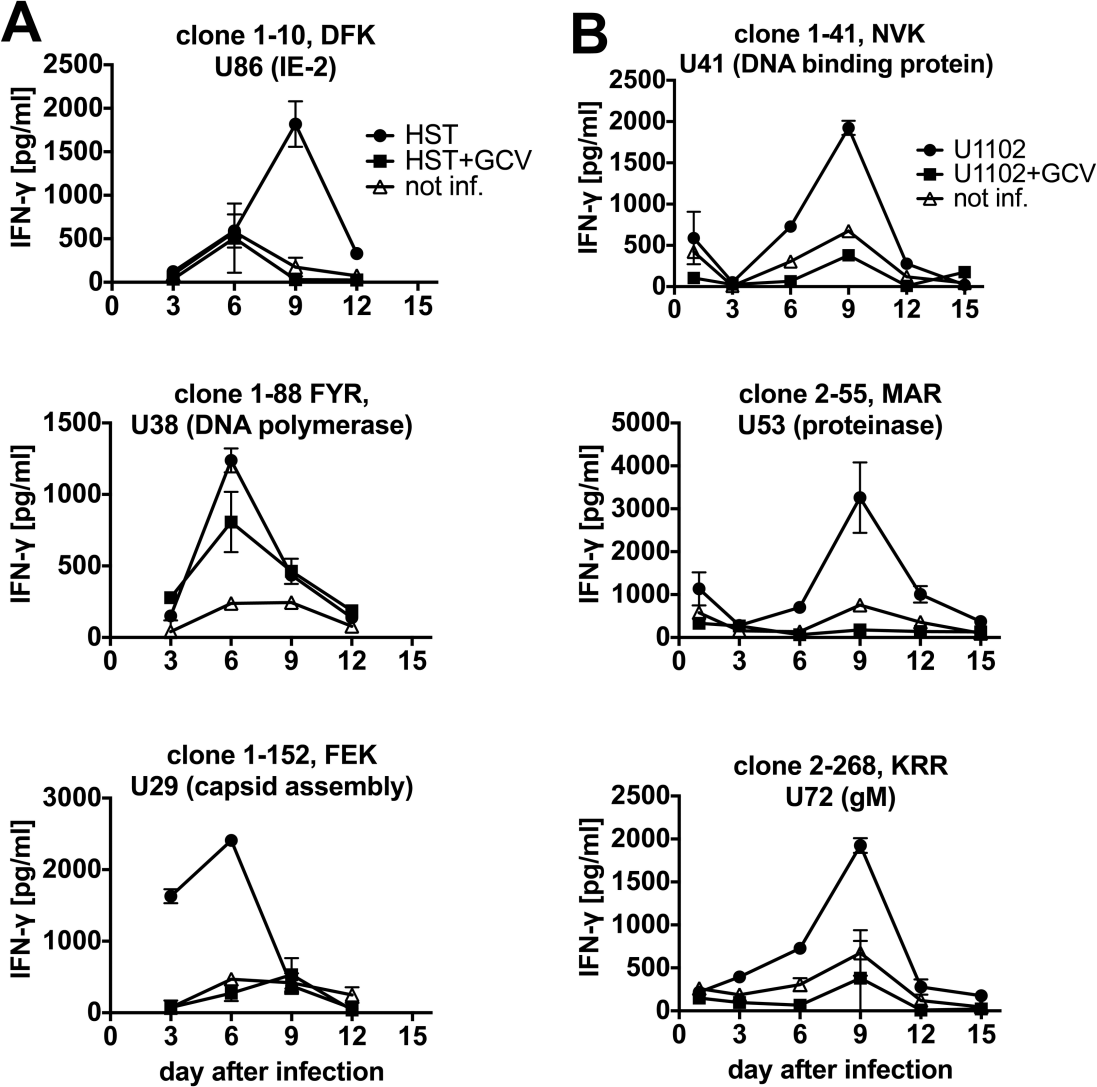
Antigen presentation by cells infected with HHV-6B and HHV-6A. HLA-B*08:01-matched PHA-activated CD4 T cells were infected with HHV-6B strain HST (panel A) or HHV-6A strain U1102 (panel B). As a further control, the viral replication inhibitor ganciclovir (GCV) was added where indicated. At the indicated time after infection, infected cells were cocultivated with T cell clones of diverse specificities. Data are shown as mean and range of duplicates from one of two experiments.

We performed time-course T cell recognition assays with T cell clones of additional specificities, including an analysis of recognition of HHV-6A and an additional control condition in the presence of ganciclovir, an inhibitor of HHV-6 replication (Fig 6). Presentation of various IE, E, and L antigens showed distinct peaks of recognition on days 3, 6, or 9. Recognition of all epitopes was partially or fully inhibited by ganciclovir.

Taken together (Fig 2), a majority of the HHV-6B peptide-specific T cell clones tested against infected cells recognized their endogenously processed target epitope in the context of infection. Epitopes from antigens of all kinetic categories (IE, early, late) and of diverse functional roles (regulation, DNA synthesis, virus assembly, structural proteins) were presented by infected cells. No particular class of antigens appeared to be excluded from presentation. At least three epitopes were from proteins with unknown function or putative proteins; our results provide evidence that ORFs including U7, U26, and B4 are translated in infected cells. Multiple epitopes were found in antigens U38 (the DNA polymerase), U41 (the major DNA-binding protein), and U86 (the transcriptional regulator IE2).

### Frequency of HLA-B*08:01-restricted HHV-6B-specific T cells in peripheral blood

**Fig 7:**
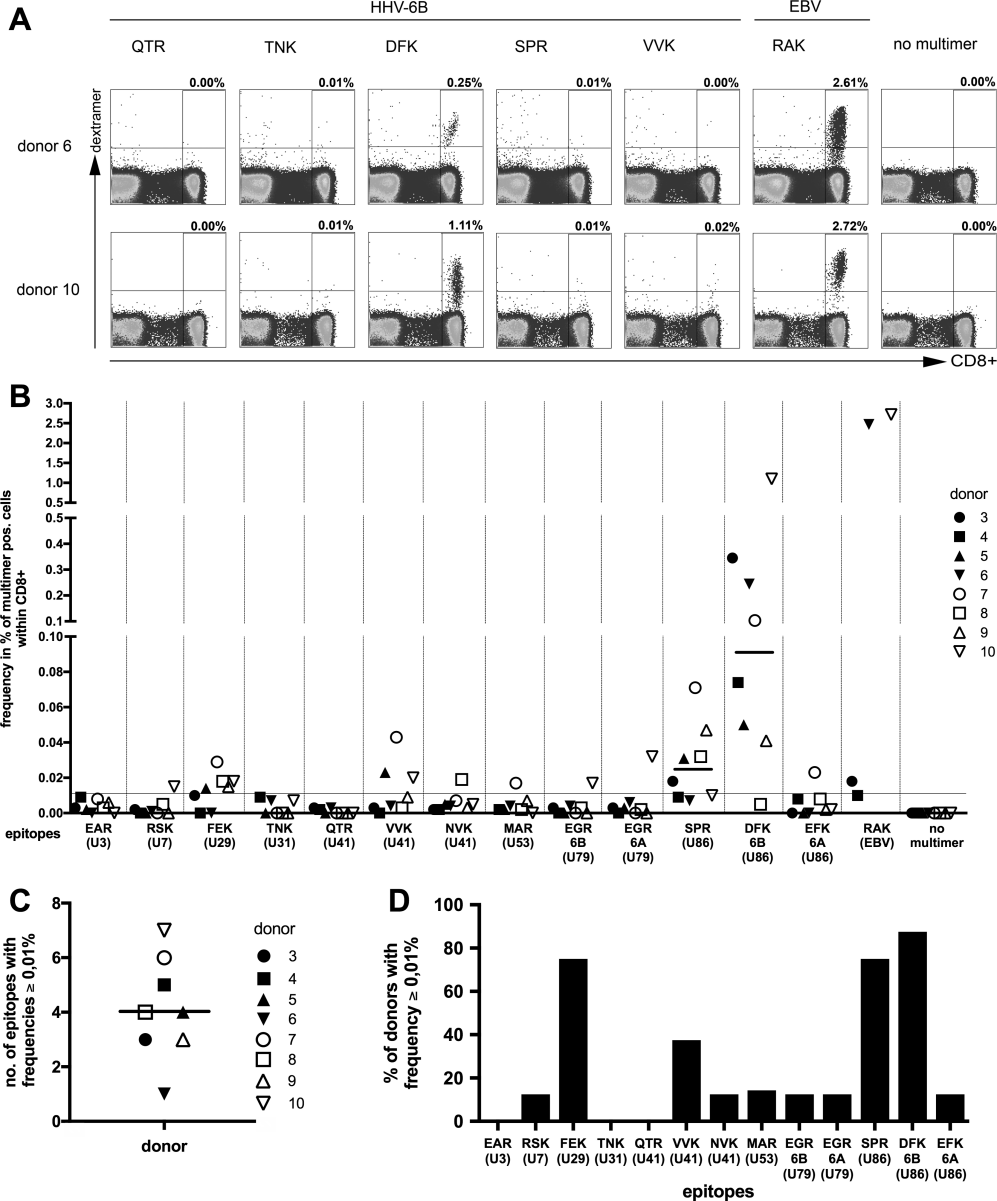
Frequencies of HLA-B*08:01-restricted HHV-6-specific T cells in peripheral blood. PBMCs from eight healthy adult HLA-B*08:01-positive donors were stained with thirteen HLA-B*08:01/peptide multimers (dextramers) carrying HHV-6 peptides as indicated. An HLA-B*08:01 dextramer carrying the EBV epitope RAK served as a control. (A) Examples of dextramer staining for two donors and six dextramers. (B) Frequencies of multimer-staining cells in eight healthy donors. Donors were known to be HHV-6-seropositive, except donor 9, whose serostatus was not known. Only four donors could be stained with RAK; donor 3 was not stained with TNK and MAR. A median of 7×10^5^ cells was stained for FACS analysis. (C) Number per donor of HHV-6B/6A multimer-staining populations that exceeded 0.01% of CD8 T cells. (D) For each HHV-6 epitope, the proportion of donors is shown who had multimer-staining populations that exceeded 0.01% of CD8 T cells.

A panel of thirteen HLA-B*08:01/peptide multimers (dextramers) was commercially synthesized. For inclusion in this panel, 11 of the 16 epitopes in their HHV-6B variants were arbitrarily chosen. For two of these epitopes, multimers loaded with their variant HHV-6A peptide were also synthesized; these included the HHV-6A variant of EGR (from U79) and the "EFK" variant of DFK (from U86; compare Fig 2). As a positive control, a multimer for the Epstein-Barr virus epitope RAKFKQLL (RAK) from the BZLF1 antigen was synthesized in parallel. All these multimers were used to stain PBMCs from eight healthy donors for analysis in flow cytometry, to determine ex vivo frequencies of HHV-6-specific CD8 T cells (Fig 7). As the examples in Fig 7A show, T cells that bound a multimer DFK/B*08:01, carrying the DFK peptide from U86, often had an elevated frequency and appeared as clearly distinct populations. T cells that bound other HHV-6 multimers were usually much less frequent. Overall, DFK-specific T cells were detectable in 7 of 8 healthy donors, with a median frequency of 0.09% of CD8+ T cells (0.005%– 1.11%). The second most frequent population were T cells specific for the SPR epitope, also from U86 (median 0.025% of CD8+ T cells; 0.007%– 0.07%). Thus, while T cells specific for many HHV-6 epitopes were in most donors not detectable above a standard baseline of 0.01%, we identified an HHV-6B epitope, DFK, that regularly allowed clear ex vivo detection of specific T cells by multimer staining. In contrast, T cells specific for the HHV-6A variant of this epitope, EFK, were of low frequency or absent. DFK-specific T cells displayed a mixed phenotype ex vivo with respect to markers of central memory, effector memory or terminal differentiation (S1 Fig).

The median number per donor of different HHV-6 epitope specificities with a frequency higher than 0.01% was four (Fig 7C), and the highest number was seven. However, this number is likely to underestimate the overall number of specificities including those of lower frequency that are present in a donor, considering that the number of different specificities in donor 1 and 2 that could be obtained as T-cell clones after specific expansion was 17 and 12, respectively (Table 2). There were three HHV-6 epitopes that elicited responses higher than 0.01% in more than half of the donors (Fig 7D). A complete set of FACS plots is provided as supporting information (S2 and S3 Figs).

### Detection of HHV-6-specific CD8 T cells in patients after allo-HSCT

**Fig 8:**
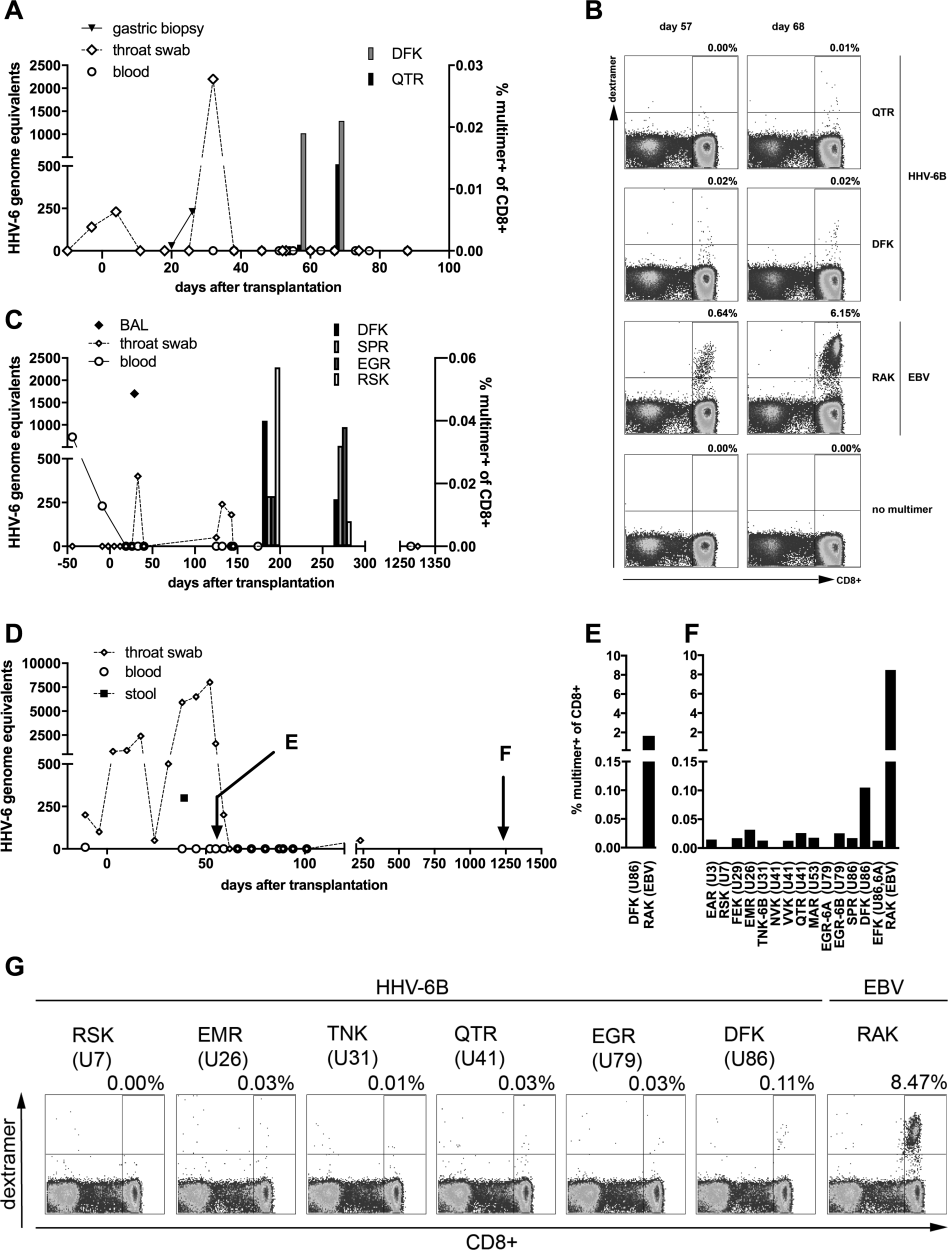
Reconstitution of HHV-6B-specific T cells in patients after allo-HSCT. (A) HHV-6 DNA and HHV-6-specific CD8 T cells in patient 1. Two peripheral blood samples (day 57, day 68) were available for staining with three HLA-B*08:01/peptide multimers, DFK and QTR from HHV-6B and RAK from EBV BZLF1. (B) Dot plots for multimer stainings of patient 1. (C) Detection of HHV-6 DNA and of HHV-6-specific CD8 T cells in patient 2. Two peripheral blood samples (day 182, day 266) were stained with HLA-B*08:01/peptide multimers carrying HHV-6B peptides DFK, SPR, EGR, and RSK. (D) Detection of HHV-6 DNA and time of HLA/peptide multimer analysis in patient 3. Peripheral blood samples were available for multimer staining from an early and a late time point (day 56, day 1221). (E) Multimer staining for detection of HHV-6-specific T cells (DFK) and EBV-specific T cells in patient 3 at day 56. (F) Multimer staining for HHV-6-specific T cells (14 epitopes as indicated) and EBV-specific T cells (RAK) in patient 3 at day 1221. (G) Exemplary dot plots of multimer stainings of patient 3 at day 1221.

We analyzed the frequency of HHV-6-specific multimer staining-positive CD8 T cells in peripheral blood of three patients after HLA-B*08:01-positive allo-HSCT from unrelated HLA-matched donors. Patient 1 had detectable HHV-6 in throat swabs at the time of transplantation. In the third to fifth week after transplantation, while in aplasia, this patient underwent an episode of HHV-6 reactivation, detectable in gastric biopsy and throat swabs, with symptoms of skin rash and nausea. Treatment with foscarnet was initiated. At day +54, EBV reactivation was detected, and was treated with cidofovir and rituximab. Probably due to viral infection, engraftment was delayed until day +105. Samples were available for analysis of specific T cells on days +57 and +68, at a time when HHV-6 reactivation had subsided (Fig 8A). DFK-specific T cells were detected at both times at similar levels, while QTR-specific T cells were increasing (Fig 8A and 8B).

Patient 2 showed HHV-6 reactivation at day +29 after allo-HSCT with detection of the virus in bronchoalveolar lavage (BAL), performed due to a CT scan showing pneumonia. A concurrent infection with Aspergillus fumigatus was found, as well as EBV and adenovirus reactivation. Patient 2 developed a histologically proven post-transplant lymphoproliferative disorder (PTLD) at day +94, and treatment with cidofovir and rituximab was performed. HHV-6-specific T cells targeting four of four different epitopes were detected in patient 2 at moderate frequencies in two of two samples after resolution of HHV-6 reactivation (Fig 8C).

Patient 3, who suffered from severe aplastic anemia, was admitted to allo-HSCT with ongoing detection of HHV-6 in throat swabs after immunosuppressive treatment consisting of anti-thymocyte globulin (ATG), corticosteroids, and cyclosporine A. No specific HHV-6-related symptoms were observed. During aplasia, a concurrent enteral adenovirus reactivation occurred, and progressed to disseminated adenovirus disease. The patient was treated with cidofovir and adenovirus-specific T cells, and fully recovered. Viral infection probably contributed to delayed engraftment. HHV-6-specific T cells could be analyzed in an early sample concurrent with ongoing HHV-6 reactivation (day +56) and a late sample (day +1221). Only DFK and the EBV epitope RAK could be studied in the early sample due to a shortage of material. DFK-specific CD8 T cells were absent at the time of reactivation (Fig 8D and 8E), but were well reconstituted at the late time point (Fig 8D, 8F and 8G). In addition, there was evidence for low-frequency establishment of CD8 T cells specific for various other HHV-6 epitopes (Fig 8F and 8G) at the late time point in this patient, who has remained alive and well until now.

Taken together, these data provide tentative evidence that reconstitution of CD8 T cells specific for HLA-B*08:01-restricted HHV-6 epitopes, notably DFK, may be associated with control of viral reactivation in patients after allo-HSCT. However, all patients were treated with cidofovir, which has high activity against HHV-6. Studies in larger patient cohorts will be necessary to establish an association of particular HHV-6 T-cell specificities and control of infection.

### Characteristics of HLA-B*08:01-restricted epitope sequences

**Fig 9:**
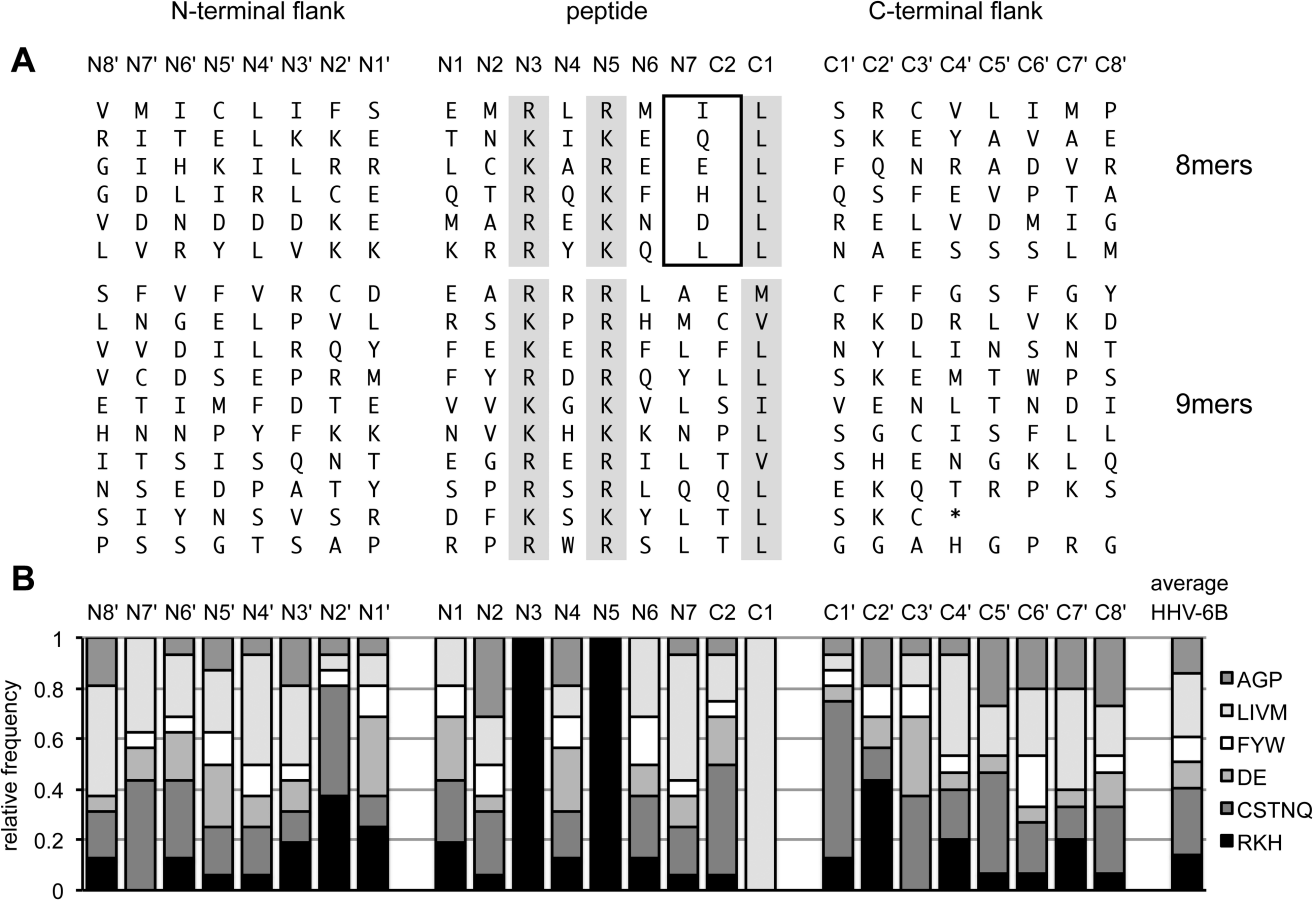
Amino acid composition of HLA-B*08:01-restricted HHV-6B epitopes and their flanking sequences. **(A)** For 16 confirmed epitopes presented by infected cells, N-terminal flanking amino acid sequences (N8' to N1'), epitope sequences (N1 to N7, C2, C1), and C-terminal flanking sequences (C1' to C8') were aligned. Predefined anchors are shaded in grey. The box indicates that the amino acids at position N7 = C2) of octameric peptides were considered twice; they were included in the calculations shown in panel B as instances of the N7 position and of the C2 position. (B) Amino acid frequency in each position, categorized into different chemical classes of amino acids. AGP, small or not otherwise categorized; LIVM, aliphatic; FYW, aromatic; DE, acidic; CSTNQ, uncharged polar; RKH, basic.

Our study identified a total of 16 HLA-B*08:01-restricted epitopes that were presented by infected cells to specific CD8 T cells, based on a set of 299 peptides as epitope candidates. This test set consisted of all HHV-6B peptides that conformed to a simplified HLA-B*08:01 motif (Table 1) defined by the presence of three anchor residues, while any amino acid was allowed in other positions of the peptide. To find out if other internal or flanking sequences were non-randomly enriched for preferred residues or motifs, we aligned our 16 epitopes and their flanking regions in their proteins of origin (Fig 9A) and analyzed their amino acid content in each position, subdividing amino acids into broad categories according to their chemical characteristics (Fig 9B).

In our set of 16 confirmed epitopes, there were nine arginines and seven lysines each in anchor positions N3 and N5, suggesting that there was no strong preference for either of these two. Each of the four permitted aliphatic residues was found in the C-terminal anchor position (C1) of the nonameric epitopes, with a preference for leucine, which may simply mirror the higher frequency of this amino acid in the viral proteome (L, 10.1%; I, 6.4%; V, 6.2%; M, 2.4% in the HHV-6B GenBank reference sequence NC_000898). All six octamers had a leucine in C1. A tendency for leucine to be enriched in N7 was noted. Other than that, there was no strong enrichment of particular amino acids within the peptide other than in the three pre-defined anchor positions, and at least five of the six chemical categories were represented in each internal non-anchor position.

Somewhat more conspicuous patterns were seen in the regions flanking the peptide. N2' was often an uncharged polar amino acid (C, S, T, N, Q) or a basic amino acid (R, K, H), C1' was often serine or another uncharged polar amino acid, and C2' was often a basic amino acid. We calculated the likelihood that such enrichments occurred by chance using Fisher's exact test, comparing the 16 epitopes to the rest of the 299 peptide candidates (Table 3). The lowest probabilities of enrichment by chance were calculated for uncharged polar or basic amino acids in position N2' (p = 0.0010), serines in C1' (p = 0.0016), polar uncharged amino acids in C1' (0.0006), and lysine in C2' (p = 0.0013). Thus, the strongest tendency in HLA-B*08:01-restricted T cell epitopes to follow conserved motifs (apart from the three pre-defined anchor residues) was not found for peptide-internal positions, but for certain flanking positions.

**Table 3:** Probabilities of randomness of the observed amino acid enrichment in selected internal or flanking positions of HLA-B*08:01 epitopes. Nomenclature of positions is as in Fig 8. Probabilities (p) were calculated by Fisher's exact test. No correction for multiple testing was performed.

position	amino acid	occurrence in epitopes	occurrence in non-epitopes		p
N7	L	6	28		0.0048
	other	10	255		
C1	L	12	108		0.0067
	other	4	175		
N2'	K	4	14		0.0107
	other	12	269		
N2'	RKH	6	35		0.0129
	other	10	248		
N2'	RKHCSTNQ	13	108		0.0010
	other	3	175		
N1'	E	4	13		0.0086
	other	12	270		
C1'	S	6	22		0.0016
	other	10	261		
C1'	CSTNQ	10	59		0.0006
	other	6	224		
C1'	RKHCSTNQ	12	104		0.0032
	other	4	179		
C2'	K	5	13		0.0013
	other	11	270		
C2'	RKH	7	39		0.0050
	other	9	244		

## Materials and methods

### Ethics statement

PBMCs from anonymized healthy adult donors were purchased from the Institute for Transfusion Medicine, University of Ulm, Germany. PBMCs from patients after allo-HSCT were obtained at the Department of Internal Medicine III, Hematopoietic Stem Cell Transplantation, Klinikum der Universität München, Munich, Germany, with written informed consent. Anonymized cord blood samples were collected at the Department of Obstetrics and Gynecology (Klinik und Poliklinik für Frauenheilkunde und Geburtshilfe, Klinikum der Universität München, Munich, Germany). The institutional review board (Ethikkommission, Klinikum der Universität München, Munich, Germany) approved these procedures (project no. 071–06–075–06, project no. 17–455).

### Cell culture, healthy donors

Standard cell culture medium was RPMI 1640 (Life Technologies/Invitrogen, Karlsruhe, Germany) supplemented with 10% FCS (Biochrom, Berlin, Germany), 100 U/ml penicillin, 100 µg/ml streptomycin (Life Technologies/Invitrogen), and 100 nM sodium selenite (ICN Biochemicals, Aurora, CO). 293T cells were cultivated in DMEM (Invitrogen) with the same supplements. Cells were all cultivated at 37°C and 5% CO2.

PBMCs were obtained by centrifugation on Ficoll/Hypaque (Biochrom). High-resolution HLA typing was performed by PCR-based methods (MVZ, Martinsried, Germany). HHV-6-specific, HLA-B*08:01-restricted T cell lines and clones were derived from four HHV-6 IgG-positive donors. Their full HLA class I types are as follows: donor 1, HLA-A*02:01, A*68:01, B*07:02, B*08:01, Cw*07:01, Cw*07:02; donor 2, HLA-A*01:01, B*08:01, B*15:01, Cw*03:03, Cw*07:01; donor 11, HLA-A*01, A*11, B*08, B*15:01, Cw*03:03, Cw*07:01; donor 12, HLA-A*01:01, A*02:01, B*08:01, B*40:01.

In healthy donors, HHV-6 IgG was determined by immunofluorescence test at Max-von-Pettenkofer Institute, Munich, Germany, with the exception of donors 3, 4, 7, and 8, in whom it was determined using the HHV-6 IgG ELISA kit (Abnova). Cell lines and cultures from these and other HLA-typed donors were used as antigen-presenting cells in T cell assays. Mini-lymphoblastoid cell lines (mLCLs) were generated by infection of PBMC with mini-Epstein-Barr viruses as described [47]. CD40-activated B-cell cultures were established as described [48] and maintained by weekly replating on irradiated (180Gy) LL8 stimulator cells in the presence of 2 ng/ml rIL-4 (R&D Systems). LL8 cells are murine L929 fibroblasts stably transfected with human CD40L [49]. Human embryonic kidney cells 293T (partial HLA type: HLA-A*02:01, B*07:02) were obtained from ATCC (CRL-11268).

### Patients

HHV-6-specific T cells were analyzed in peripheral blood samples from three adult patients after allo-HSCT. Transplant indication was severe aplastic anemia (SAA) in patients 1 and 3, and acute myeloid leukemia (AML) in patient 2. G-CSF-mobilized peripheral blood stem cells from an HLA-matched unrelated donor were used in patients 1 and 2; bone marrow donated by an HLA-matched unrelated donor was used in patient 3. GvHD prophylaxis consisted of cyclosporine A plus sirolimus (n = 2) or mycophenolate mofetil (n = 1). All patients and donors were HLA-B*08:01-positive and CMV-seronegative. Patients received standard antiviral prophylaxis with acyclovir. Viral infection/reactivation was monitored weekly by quantitative PCR in peripheral blood, including HHV-6. Other specimens like stool, urine and throat swab samples were monitored for virus reactivation weekly on a routine basis as indicated. A detailed overview of the characteristics of patients, donors, and transplant procedures is provided (Supporting S2 Table).

### Peptides

Peptide sequences adhering to the HLA-B*08:01 anchor motif were extracted from the HHV-6B strain Z29 reference sequence (GenBank NC_000898) using the text editor Tex-Edit Plus and a script in the AppleScript language. The 299 peptides of the HLA-B*08:01 candidate library were synthesized by JPT (Berlin, Germany) in a "Research Track" format. Each peptide was analyzed by liquid chromatography–mass spectrometry. Median purity of peptides was 77%. Nineteen peptides had a purity below 50% (minimum 25.5%), none of these was recognized by any T cell clone. Peptides were reconstituted in 100% dimethyl sulfoxide (DMSO) and stored at –20°C. DMSO concentration in all T cell effector assays was kept below 0.1% (vol/vol).

### Generation of HHV-6-specific T cell lines and clones

PBMCs from HHV-6-positive donors were enriched for HHV-6B-specific T cells by stimulation with a mix of 146 octameric peptides or 153 nonameric peptides that represent HLA-B*08:01 candidate epitopes from HHV-6B, using a protocol employing autologous CD40-activated B cells. For peptide loading, PBMC (first stimulation) or CD40-activated B cells (all later stimulations) were coincubated with octamer or nonamer peptide pool (1 µg/ml for each peptide) at 37°C for 2 h, and washed three times with PBS.

The T cell stimulation protocol was initiated by peptide loading of PBMC, which were then plated at 5×10^6^ cells in 2 mL per well of a 12-well plate. Ten to 14 days later, cells were pooled, counted using trypan blue staining, and restimulated at 3×10^6^ cells in 2 mL medium per well with freshly irradiated (50 Gy) autologous CD40-activated B cells, previously loaded with peptides, to reach an effector:stimulator ratio of 4:1, in the presence of 25–50 U/mL recombinant IL-2 („Proleukin S“, Novartis). Cells were restimulated every following week with peptide-loaded CD40-activated B cells in the same manner, with the exception that the IL-2 concentration was successively increased to 100 U/mL. Between stimulations, the T cell cultures were expanded using fresh IL-2-containing culture medium as seemed necessary, judging from the visual appearance of the cultures. For cytotoxicity analysis, PBMCs were stimulated with a single peptide (DFK from U86) and autologous CD40-activated B cells in an analogous manner, and tested at day 29 of cultivation.

For single cell cloning of polyclonal T cell cultures, 0.7 or 2.5 T cells/well were seeded into 96-well round-bottom plates, together with 2×10^4^/well irradiated (50 Gy) HLA-B*08:01-positive mini-LCLs loaded with the octameric or nonameric HHV-6B peptide pool, 3×10^5^ cells/well of a mixture of irradiated (50 Gy) allogeneic PBMCs from three donors, and 1000 U/mL IL-2. Outgrowing T cell clones were expanded in 96-well round-bottom plates by restimulating every 2 weeks under equivalent conditions. Later, clones with known peptide specificity were restimulated in an analogous manner but using only the single specific peptide.

### Flow cytometry

HLA/peptide multimers in the form of phycoerythrin-(PE)-labeled HLA-B*08:01/peptide dextramers were purchased from Immudex, Copenhagen, Denmark. Dextramers contained one of thirteen HHV-6 peptides or the peptide RAK (full sequence RAKFKQLL) from the BZLF1 protein from Epstein-Barr virus. Dextramers covered the epitopes EAR, RSK, FEK, QTR, VVK, NVK, MAR, whose peptide sequences are identical in HHV-6A and HHV-6B; the epitopes TNK, EGR-6B and DFK from HHV-6B, which differ from their HHV-6A counterparts in one to three amino acids; the epitopes EGR-6A and EFK from HHV-6A (EFK being the HHV-6A version of DFK); and the HHV-6B epitope SPR, which has no HHV-6A counterpart. The sequences of HHV-6 peptides are provided in Fig 2.

For quantification of antigen-specific CD8+ T cells in peripheral blood from healthy donors using dextramers, a median of 7x10^5^ PBMCs per staining was treated as follows. Cells were stained for 10 minutes at room temperature with 1 μl PE-labeled HLA/peptide dextramer. For negative controls, cells were processed identically, but dextramer was not added. After washing with PBS supplemented with 2% FCS, cells were counterstained on ice for 15 minutes with anti-CD4-FITC (clone RPA-T4), anti-CD3-PE-Cy5 (clone HIT3a), and anti-CD8-APC (clone RPA-T8) antibodies (all BioLegend). Cells were then washed with PBS/FCS and resuspended in 1.6% formaldehyde (Carl Roth) in PBS for fixation, stored at 4°C, and analyzed within one day on a Becton Dickinson FACSCalibur flow cytometer. Data analysis was performed using FlowJo 9.5.3 software (Tree Star): lymphocytes were gated in a forward/sideward scatter dot plot, then CD3+CD4– cells were analyzed for the proportion of multimer-positive cells within CD8+ T cells.

For healthy donors 4, 6, 10 and transplantation patients, a variation of this protocol was used. Instead of anti-CD4-FITC, a "dump channel" mix of FITC-labeled antibodies anti-CD14 (clone TÜK4, Miltenyi Biotec), anti-CD19 (clone LT19, Miltenyi Biotec), and anti-TCR-γδ (clone 5A6.E9, Life Technologies) was used. Viable lymphocytes were gated according to forward/sideward scatter, and FITC-positive cells were excluded. For patient samples, only 3x10^5^ PBMCs were used per staining.

A range of differentiation markers was analyzed on DFK-specific T cells in donor 3 and 6. Staining with dextramer DFK was combined with FITC-labeled anti-CD14 and anti-CD19 antibodies as above (dump channel) and CD3-Alexa Fluor 700 (clone HIT3a, BioLegend); additional antibodies in panel A were CD8-APC (clone RPA-T8), CCR7-PE-Cy7 (clone G043H7), and CD45RA-Pacific Blue (clone HI100; all BioLegend); additional antibodies in panel B were CD8-APC-H7 (clone SK1, BD), CD27-APC (clone O323, BioLegend), CD28-PE-Cy5 (clone CD28.2, BD Pharmingen), and CD57-Pacific Blue (clone HCD57, BioLegend).

Dot plots displaying flow cytometry data in Figs 7 and 8, S2 and S3 Figs span, in both dimensions, a range from 1 to 10000 arbitrary fluorescence units in a logarithmic scale. Data in S1 Fig are presented in a biexponential scale spanning a range from 10^−3^ to 10^5^ arbitrary units in both dimensions.

### T cell effector assays

To verify the HLA restriction of HHV-6B-specific T cell clones, 293T cells were transfected with a HLA-B*08:01 expression plasmid (kindly provided by Josef Mautner, Munich) by calcium phosphate precipitation. Twenty-four hours later, cells were harvested, washed with PBS, loaded with single peptides at 1 µg/ml, washed three times, and used as targets in T cell assays.

HHV-6B-specific T cell lines and T cell clones were analyzed for antigen-specific IFN-γ secretion in ELISA or ELISPOT assays. Effector cells (10^4^, unless noted otherwise) were cocultivated overnight (16–18 h) with target cells (2x10^4^, unless noted otherwise) in 200 μL per well of a 96 V-well plate at 37°C and 5% CO_2_. Then supernatants were harvested, and an IFN-γ ELISA was performed according to the manufacturer’s protocol (Mabtech, Nacka, Sweden).

IFN-γ ELISPOT assays were used to determine the frequency of specific T cells in freshly isolated PBMCs and polyclonal T cell lines. They were performed according to the reagent manufacturer’s protocol (Mabtech, Nacka, Sweden) in 96-well MultiScreen-HA plates (Millipore) in 200 μL medium per well, with an overnight incubation period of 16–18 hours at 37°C and 5% CO_2_. To analyze PBMCs, 250,000 cells were distributed to each well and directly loaded with antigenic peptide. To analyze T cell lines, autologous CD40-stimulated B cells were loaded with antigenic peptides, washed, and co-incubated at 5x10^4^/well together with the T cells at 10,000 cells/well. Spots were developed using the AP Conjugate Substrate Kit from Bio-Rad. Spots were counted in an automated ELISPOT reader (CTL).

To determine the cytotoxic activity of HHV-6B-specific T cells against HHV-6B-infected cells, calcein release assays were performed. HHV-6B-infected target cells (see below) were loaded with Calcein AM (5 μg/ml, Molecular Probes) for 30 min at 37°C in standard medium. Cells were washed three times and resuspended in RPMI medium without phenol red supplemented with 5% FCS. Effector T cells were washed once and resuspended in the same medium. T cells and target cells were combined in V-bottom 96-well plates (200 μl total volume per well), with 5,000 target cells per well and 5,000–80,000 T cells per well (effector: target ratio 1:1 to 16:1), in four replicates of each condition. After 3.5 hours at 37°C and 5% CO_2_, supernatants (100 μl per well) were collected, and fluorescence was measured (excitation 485 nm, emission 535 nm). Specific lysis was calculated relative to maximal lysis (100%, targets incubated with 0.5% Triton X-100) and minimal lysis (0%, targets incubated in the absence of T cells).

### HHV-6A and -6B propagation and infection

HHV-6A (strain U1102) and HHV-6B (strain HST) were purchased from NCPV, UK, and serially propagated on phytohemagglutinine (PHA)-activated cord blood mononuclear cells. Fresh or cryoconserved cord blood cells at 2x10^6^ cells in 2 ml per well of a 24-well plate were stimulated with 5 μg/ml PHA-M (Calbiochem). Three days later, cells were infected with virus suspension from previous passages (230 μl/well). After 5–7 days, when the cytopathic effect appeared maximal, cell cultures were harvested, cells were pelleted by centrifugation at 300 *g* for 10 min, and supernatants were stored in aliquots at -80°C.

Peripheral blood cells from adult donors with known HLA types were used to prepare HHV-6A/B-infected target cells for the analysis of T cell recognition. CD4 T cells were positively isolated from PBMC using anti-CD4-coupled paramagnetic beads (Miltenyi Biotec), and 2x10^6^ CD4+ cells were activated in 2 mL per well of a 24-well plate using 5 μg/mL PHA. After 3 days, the cells were pooled, counted, replated at 2x10^6^ cells/well, and infected with 230 μl/well of HHV-6A or HHV-6B virus stocks. Thereafter, infected T cell cultures were resupplied with fresh medium every 3 days on average. At different time points after infection, cells were used as targets for HHV-6-specific T cell clones in cytokine secretion assays. At every time point, infected cells were harvested, washed and counted in Trypan Blue solution immediately before they were combined with HHV-6-specific T cells at constant numbers (10^4^ effector T cells, 2 × 10^4^ infected cells or control targets). In selected experiments as indicated, ganciclovir (20 μg/mL, Roche) was added immediately before infection.

## Discussion

Here we present a cross-sectional analysis of the CD8 T cell response to HHV-6, and an overview of antigens recognized by this response. For one exemplary HLA class I molecule, HLA-B*08:01, we identified candidate epitopes all across the HHV-6B proteome, and tested which of these represent bona fide epitopes. A large set of T cell clones was established to assess and correlate epitope specificity and antiviral reactivity with precision. Frequencies of specific T cells in healthy donors and allogeneic transplant patients were determined by multimer staining. A majority of peptides against which we could raise T cells were presented by infected cells, and epitopes from all classes of viral antigens were presented. Ex vivo frequencies of specific T cells were low for most epitopes. However, U86-specific T cells were readily detectable ex vivo in most donors and patients. U86 is thus a candidate for an immunodominant CD8 T cell antigen of HHV-6. Moreover, we describe the presence of HLA-B*08:01-restricted HHV-6-specific T cells in patients who were able to control episodes of HHV-6 reactivation after stem cell transplantation.

Taken together, the present work provides a cross-sectional overview of the structure of the HHV-6-specific CD8 T cell response at two levels. It shows that multiple viral antigens of different functional and kinetic classes furnish epitopes for T cell recognition; and it describes the quantitative contributions of the different specificities to the T cell repertoire, including identification of a prominent antigen.

Our study extends earlier investigations of the HHV-6-specific CD8 T cell response that were limited to the analysis of responses to five pre-chosen proteins: four virion proteins (U11, U14, U54, U71) and the IE-1 transactivator U90 [18–21]. The motivation to choose those antigens was their correspondence to immunogenic proteins of human CMV. The present work employed a method that was independent of such criteria and targeted CD8 T cell epitopes across the viral proteome. HLA-B*08:01-restricted epitopes were identified in 12 proteins of varied function and from all phases of the viral replication cycle. No epitope was derived from any of the five antigens studied earlier, although 12 candidates from these proteins were included in our analysis. This suggests that immunity to HCMV antigens has limited power to predict the specificity of CD8 T cell responses to HHV-6. We cannot exclude, however, that CD8 T cells that target additional epitopes, including such from the five proteins mentioned, may exist in the T cell repertoire. Of note, U86 attracted the strongest T cell responses among the antigens described here, and its CMV counterpart IE-2/UL122 is a strong CD8 T cell antigen [25]. This suggests that certain commonalities between recognition patterns of CMV and HHV-6 antigens may exist.

However, the overall composition and diversity of the HHV-6-specific CD8 T cell repertoire, as characterized here, appears to stand in marked contrast to the best-studied herpesviruses, CMV (a β-herpesvirus like HHV-6) and EBV (a γ-herpesvirus). Contrary to HHV-6, CMV elicits very large CD8 T cell responses, amounting to an average of 10% of the peripheral CD8 T cell repertoire of healthy carriers [25]. HLA-B*08:01-restricted T cells make a strong contribution to this response [23,50]. EBV-specific CD8 T cells account for a smaller proportion of total CD8 T cells in healthy donors [51], but, for example, the HLA-B*08:01-restricted RAKFKQLL epitope (Table 2) is recognized by a median of about 2% of CD8 T cells, and frequencies above 5% are no rarity [29,52]. CD8 T cell responses to CMV further increase in the elderly [53,54], and EBV-specific CD8 T cells are strongly elevated in patients with symptomatic primary EBV infection [55]. On the other hand, the diversity of the CD8 T cell response to CMV or EBV appears restricted. For example, the database IEDB [56] currently lists only three HLA-B*08:01-restricted epitopes from CMV and four from EBV, counting strain variants as one epitope. In CMV carriers, a median of eight out of 213 ORFs is recognized by CD8 T cells [25], and a majority of EBV antigens appear exempt from CD8 T cell recognition [51]. Thus, it appears that the diversity of epitopes and antigens available for presentation by infected cells is distinctly larger in HHV-6 than in the two paradigmatic human herpesviruses. However, it cannot be excluded that more diverse repertoires of low-frequency CD8 T cell specificities in EBV or CMV have so far escaped detection, possibly because their responses were masked by more dominant CD8 T cell populations.

In contrast, CD8 T cells specific for other herpesviruses such as varicella-zoster virus (VZV) or herpes simplex virus (HSV-1) are maintained at relatively low frequencies in healthy carriers [57–59]. In HSV-1, CD8 T cells appear to target multiple antigens from different phases of infection, whereas IFN-γ responses to individual epitope peptides ex vivo have frequencies of 1 in 10^4^ PBMCs or lower. A large number of potential epitopes from HSV-1 were described, with up to 13 sharing the same HLA class I restriction [57], although it is not clear yet whether a majority of these is presented by infected cells. This structure of the T-cell repertoire appears comparable to the one described here for HHV-6. Less is known about the VZV CD8 epitope repertoire, but available data are compatible with a highly diverse repertoire which is in part shaped by cross-reactivity of CD8 T cells to HSV and VZV [60].

Potential reasons for differentially structured antiviral CD8 T cell repertoires may be sought in the patterns of cellular tropism of these viruses. CMV resides latently in monocytes and myeloid precursors and is reactivated upon their differentiation to dendritic cells [61], whereas EBV infects B cells in diverse activation states [62]. Infection of professional antigen-presenting cells by CMV and EBV may favour competitive clonal expansion and selection of immunodominant T cells into the repertoire [63,64]. In other herpesviruses, tropism for professional antigen-presenting cells is less predominant [5,65]—although HHV-6 was shown to infect monocytes and other antigen-presenting cells in vivo [5]. Also, it appears that the repertoire of viral immunoevasive molecules that directly interfere with steps in the HLA class I presentation pathway is larger for CMV or EBV [49,66,67] than for other herpesviruses [58,68]. Co-expression of many immunoevasive functions in CMV and EBV may limit the number of epitopes that escape such regulatory mechanisms [69,70], and this may lead to competitive advantage and immunodominance of T cells that recognize their epitopes in the context of infection.

Our analysis of T cell epitopes was limited to only one allotype, HLA-B*08:01, and extrapolations to the CD8 T cell repertoire in general must be made with caution. More comprehensive studies on the entire CD8 T cell repertoire to HHV-6 will be necessary to strengthen our present suggestions. However, available information on CD8 T cell responses to other complex viruses indicates that HLA-B*08:01, wherever studied, rarely fails to be an effective presenter of epitopes, as shown by the examples in Table 1.

The groove of MHC class I molecules accomodates peptides for presentation to CD8 T cells [71–73]. Particularly important for stable binding are certain anchor residues [74] whose side chains reach into dedicated pockets in the peptide-binding groove. Allelic variants of MHC class I demand anchor residues required for peptide binding that can differ in their chemical nature and their position in the peptide [27,33,74]. Our identification of HLA-B*08:01-restricted T cell epitopes consisted in a functional screen of all HHV-6B-derived peptides that contained a motif of three required anchor residues [27,75], as depicted in Table 1, while any amino acid was permitted in other positions of the peptide. Application of such a simple anchor-motif based algorithm (SAMBA) is supported by the observation that a majority of well-characterized, independently verified, and potent CD8 T cell epitopes from infectious pathogens perfectly adhere to this motif, whereas amino acid usage in all other positions is more variable (see Table 1 and the references therein). Full conformity to this motif was also shown for abundant self-derived peptides eluted from HLA-B*08:01 in a seminal study [75].

Subsequent studies have, however, increasingly identified B*08:01-binding self peptides that partially deviated from the motif [76,77]. In these cases, peptides were eluted from cells co-expressing several HLA class I molecules, and their HLA-B*08:01 restriction was retrospectively inferred from the peptide sequence. Deviation from the classical motif was also observed when predicted B*08:01-restricted epitopes were validated in ex vivo ELISPOT assays with blood cells loaded with peptide [78]. However, such approaches carry the risk of identifying responses to peptides that are not endogenously processed [26] or not presented by the predicted HLA allotype [69], if those aspects are not independently tested.

Prediction of MHC class I epitopes currently relies on machine-learning algorithms trained on ever increasing datasets of MHC binders or epitopes [79,80]. To the extent that such datasets may contain a growing number of candidates whose HLA restriction and qualities as epitopes have not been verified, further progress in predicting optimal epitopes may be difficult to achieve. Therefore, in our view it is as important as ever to rigorously verify HLA restriction and endogenous presentation, optimally with target cells infected with the pathogen of interest. Reliance on T cell clones increases the accuracy of epitope identification and validation, since this ensures that the very same T cells recognize peptide and infected cells, and minimizes the likelihood of accidental cross-reactivities. Peptide-based functional screens of epitope candidates ex vivo have been successful in identifying CD8 T cell reactivities that were later confirmed to be viral epitopes, for example in CMV [23,54], but such approaches are likely to be less robust when proportions of specific T cells are low, such as for HHV-6.

This study identified sixteen HLA-B*08:01-restricted HHV-6 epitopes–defined as peptides presented by infected cells–out of 299 candidates. We took advantage of this dataset to compare amino acid usage in internal and flanking sequences of epitopes and non-epitopes. In the C-terminal anchor position (C1), Leu appeared to be favoured among admitted aliphatic residues, clearly so in octameric epitopes. Leu was also enriched in the C2 position. Otherwise, no restrictions of amino acid usage in non-anchor positions in HHV-6 epitopes were apparent, which is in line with the idea of distinct functional roles for anchors and non-anchors, and retrospectively supported our use of a SAMBA approach.

However, stronger enrichment of certain amino acids was found in peptide-flanking positions (N2', C1', and C2'). The C terminus of most MHC I ligands is generated by the proteasome [81]. Cut site preferences of human proteasomes have been identified by in vitro digestion of model proteins [82–85], and coincide well with the requirements of many MHC I allotypes (such as HLA-B*08:01) to bind peptides with a bulky hydrophobic residue in the C-terminal position. Downstream of the cut site, amino acid preferences partially diverge between model proteins [82–85]. We found uncharged hydrophilic amino acids, particularly Ser, to be enriched in the C1' position (called P1' in analyses of proteasome function). Ser in this position was also enriched after degradation of HIV Nef by the constitutive proteasome [83] and of prion protein by the immunoproteasome [84]. Increased frequency of Arg [83,85] and depletion of bulky hydrophobic amino acids [82,83,85] also agreed with our findings, whereas enrichment of Ala or Pro [82–85] did not. Although limited in size, our dataset suggests an influence of amino acid identity in C1'/P1' on effective proteasomal processing of HHV-6 epitopes. In the C2'/P2' position, we observed basic amino acids to be enriched; no clear tendency in that regard is found in the literature [84,85].

Formation of the N terminus of MHC I ligands is in many cases a complex multistep process comprising proteasomal degradation, processing by cytosolic aminopeptidases, TAP-mediated transport to the ER, and final trimming by ER aminopeptidases [86]. Nontheless, an N-terminal processing motif of MHC I ligands could be defined [86]. Consistent with our findings, this motif has the basic amino acids Lys and Arg somewhat enriched in the N2' position [86]. Basic amino acids in N-terminal overhangs of MHC I ligand precursors may favour processing by cytosolic or ER aminopeptidases [86], although the ER peptidase ERAP1 does not appear to have this preference [87]. Moreover, basic amino acids close to the N terminus of MHC I ligand precursors may support effective TAP-mediated transport to the ER [88]. Thus, amino acid usage in regions flanking HHV-6 epitope peptides is compatible with some of the described N- and C-terminal MHC I processing preferences. However, such motifs represent tendencies rather than strict criteria, so improving epitope prediction by considering processing motifs remains difficult [79]. Nontheless, we speculate that simplified peptide-flanking motifs may be useful to design screening approaches that prioritize efficiency over completeness.

Identification of multiple CD8 target antigens and epitopes as undertaken here will advance immune monitoring and immunotherapy of HHV-6. Since our study identifies an epitope (DFK from U86) that allowed detection of specific T cells (sometimes at high frequencies of up to 1.1%) in 7/8 healthy carriers and 3/3 patients after allo-HSCT, multimer staining based on this epitope will be a convenient tool for monitoring and monospecific approaches to antiviral T cell therapy [89,90]. HHV-6-specific T cell transfer after allo-HSCT is attractive and feasible. In patients who received allo-HSCT, HHV-6 reactivation and disease is associated with a lack of virus-specific T cells [10,91] and the use of transplantation procedures that lead to imperfect T cell reconstitution [92]. In a first clinical application of HHV-6-specific T cell transfer to allo-HSCT patients, T cells specific for U11, U14, and U90 were part of a protocol that employed multivirus-specific peptide-stimulated T cells derived from the transplant donor [12]. In two patients, HHV-6 reactivation was cleared after transfusion of multivirus-specific T cells, in connection with an emergence of HHV-6-specific T cells in peripheral blood [12]. Partial remissions of HHV-6 infection were also observed in a third-party approach based on similarly prepared T cells [93]. These promising initial results encourage further application and development of HHV-6-specific adoptive immunotherapy. Since multiple epitopes are targeted by HHV-6-specific CD8 T cells, a multiepitope approach [94] may be particularly promising for selection of effective HHV-6-specific T cells for immunotherapy. If TCR-transgenic T cell therapy [95] is considered, HHV-6 antigens from diverse functional classes may be suitable targets.

## Supporting information

S1 Table Full list of 299 candidates for HLA-B*08:01-restricted T cell epitopes from HHV-6B, strain Z29. (XLSX)

S2 Table Characteristics of allo-HSCT patients, donors, and transplantation procedures. (PDF)

S1 Fig Analysis of differentiation markers on HHV-6B-specific T cells ex vivo. PBMCs were stained with dextramer DFK/B*08:01, carrying the DFK peptide from U86, and differentiation markers were analyzed on gated DFK-specific CD8 T cells (CD8+, DFK+). Differentiation markers included CCR7, CD45RA (panel A), CD27, CD28, and CD57 (panel B). The dextramer-negative CD8 T cell population is shown for comparison (CD8+, DFK–).
(TIFF)

S2 Fig Dot plots of dextramer staining of PBMCs of healthy donors, part 1 (epitopes EAR, RSK, FEK, TNK, QTR, VVK, NVK, MAR). (TIF)

S3 Fig Dot plots of dextramer staining of PBMCs of healthy donors, part 2 (epitopes EGR-6B, EGR-6A, SPR, DFK, EFK, RAK, and negative control). (TIF)
