# Supplementary material for: Cross-sectional analysis of CD8 T cell immunity to human herpesvirus 6B
Source: PLoS Pathog. 2018 Apr 26;14(4):e1006991. doi: 10.1371/journal.ppat.1006991 (PMC5919459; doi:10.1371/journal.ppat.1006991)
Supplement: S2 Table — (PDF) [file ppat.1006991.s002.pdf]

S2 Table: Characteristics of allo-HSCT patients, donors, and transplantation procedures.

Characteristics	Patient 1	Patient 2	Patient 3
Patient age, years	30	43	18
Patient sex	female	female	female
Underlying disease	SAA	AML	SAA
Donor sex	male	female	male
CMV serostatus			
Donor / Recipient	neg / neg	neg / neg	neg / neg
Donor type	unrelated	unrelated	unrelated
HLA match	matched	matched	matched
Graft source	PBSCs	PBSCs	BM
Conditioning regimen	RIC	RIC	RIC
GvHD prophylaxis	ATG-CyA-Sir	ATG-CyA-MMF	ATG-CyA-Sir
Neutrophil engraftment, day	105	23	60
aGvHD	grade II	grade I	none
aGvHD onset, day	115	14	-
HHV-6 treatment	foscarnet, cidofovir	none	none
Viral coinfection	EBV	EBV, adenovirus	adenovirus

Abbreviations: neg, negative; SAA, severe aplastic anemia; AML, acute myeloid leukemia; HLA, human leukocyte antigen; PBSCs, peripheral blood stem cells; BM, bone marrow; RIC, reduced intensity conditioning; ATG, anti-thymocyte globulin; CyA, cyclosporine A; Sir, Sirolimus; aGVHD, acute graft-versus-host disease; EBV, Epstein-Barr virus.
