# Supplementary figures and images for: Cross-sectional analysis of CD8 T cell immunity to human herpesvirus 6B
Source: PLoS Pathog. 2018 Apr 26;14(4):e1006991. doi: 10.1371/journal.ppat.1006991 (PMC5919459; doi:10.1371/journal.ppat.1006991)

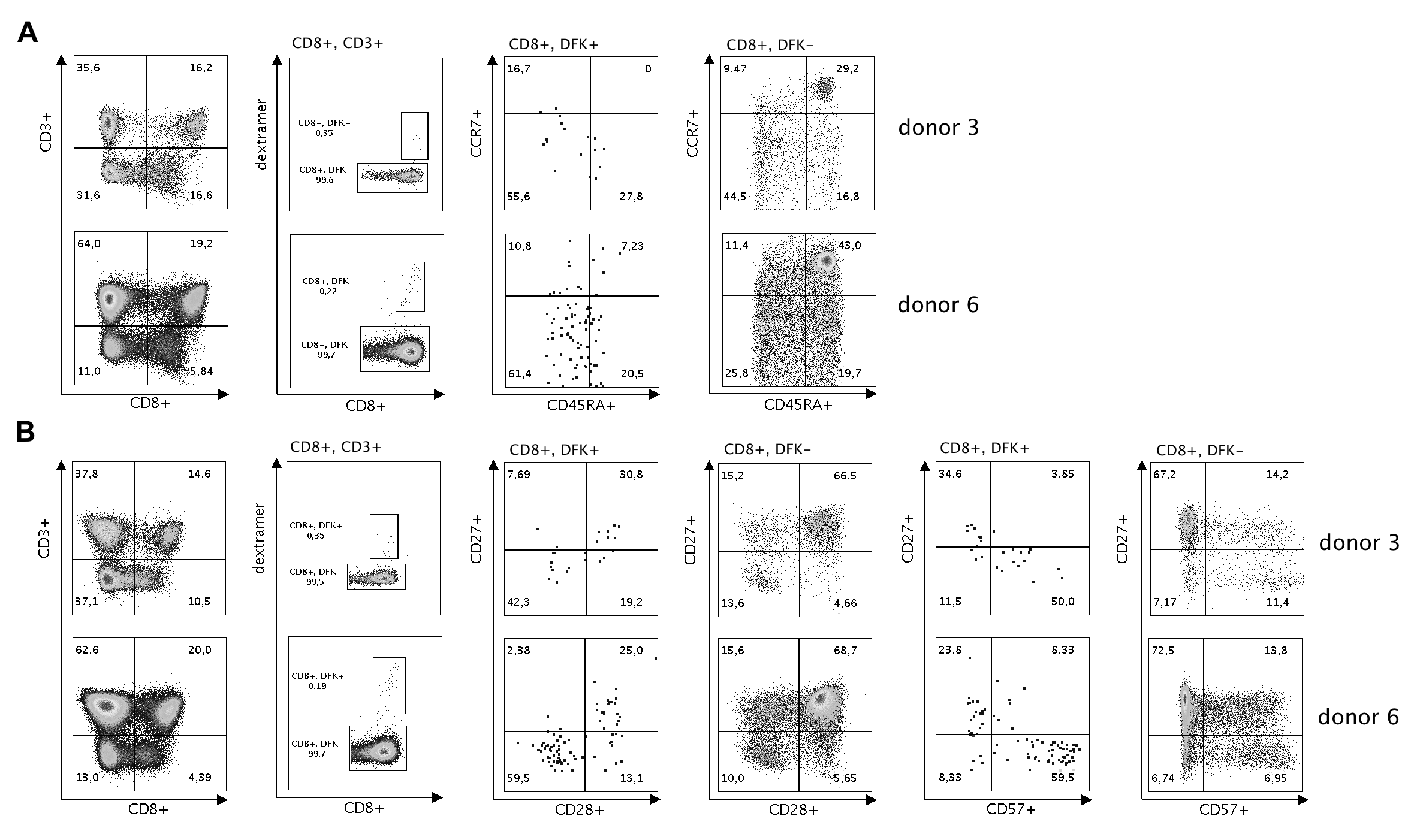

Supplement: S1 Fig — PBMCs were stained with dextramer DFK/B*08:01, carrying the DFK peptide from U86, and differentiation markers were analyzed on gated DFK-specific CD8 T cells (CD8+, DFK+). Differentiation markers included CCR7, CD45RA (panel A), CD27, CD28, and CD57 (panel B). The dextramer-negative CD8 T cell population is shown for comparison (CD8+, DFK–). (TIFF) [file ppat.1006991.s003.tiff]

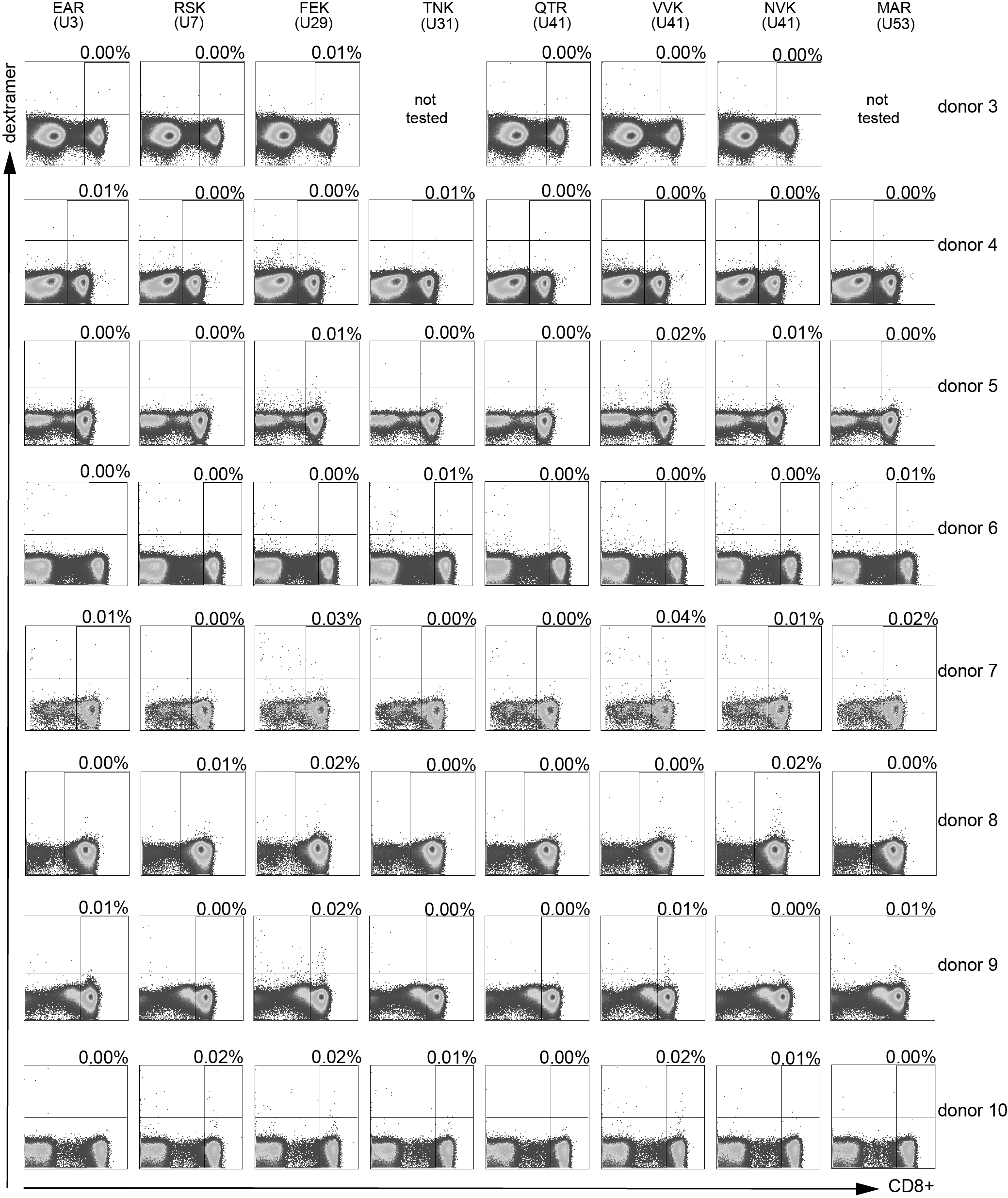

Supplement: S2 Fig — (TIF) [file ppat.1006991.s004.tif]

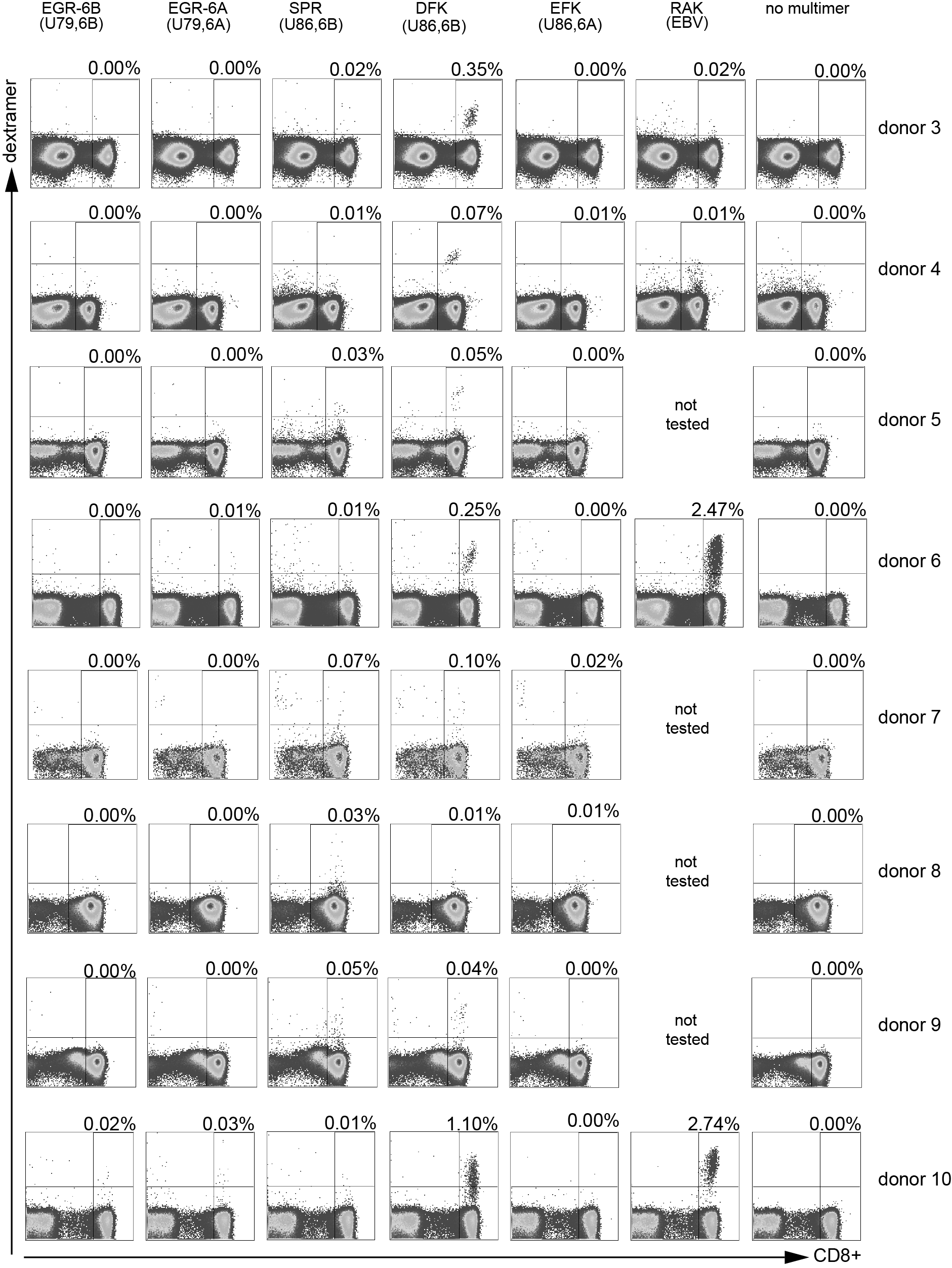

Supplement: S3 Fig — (TIF) [file ppat.1006991.s005.tif]
